# The parasitic worm product ES-62 protects the osteoimmunology axis in a mouse model of obesity-accelerated ageing

**DOI:** 10.3389/fimmu.2022.953053

**Published:** 2022-08-29

**Authors:** Margaret M. Harnett, James Doonan, Felicity E. Lumb, Jenny Crowe, Roel Olde Damink, Geraldine Buitrago, Josephine Duncombe-Moore, Debbie I. Wilkinson, Colin J. Suckling, Colin Selman, William Harnett

**Affiliations:** ^1^ Institute of Infection, Immunity and Inflammation, University of Glasgow, Glasgow, United Kingdom; ^2^ Strathclyde Institute of Pharmacy and Biomedical Sciences, University of Strathclyde, Glasgow, United Kingdom; ^3^ Institute of Medical Sciences, University of Aberdeen, Aberdeen, United Kingdom; ^4^ Department of Pure and Applied Chemistry, University of Strathclyde, Glasgow, United Kingdom; ^5^ Glasgow Ageing Research Network (GARNER), Institute of Biodiversity, Animal Health and Comparative Medicine, University of Glasgow, Glasgow, United Kingdom

**Keywords:** ageing, B lymphocyte, ES-62, inflammation, obesity, osteoimmunology

## Abstract

Despite significant increases in human lifespan over the last century, adoption of high calorie diets (HCD) has driven global increases in type-2 diabetes, obesity and cardiovascular disease, disorders precluding corresponding improvements in healthspan. Reflecting that such conditions are associated with chronic systemic inflammation, evidence is emerging that infection with parasitic helminths might protect against obesity-accelerated ageing, by virtue of their evolution of survival-promoting anti-inflammatory molecules. Indeed, ES-62, an anti-inflammatory secreted product of the filarial nematode *Acanthocheilonema viteae*, improves the healthspan of both male and female C57BL/6J mice undergoing obesity-accelerated ageing and also extends median lifespan in male animals, by positively impacting on inflammatory, adipose metabolic and gut microbiome parameters of ageing. We therefore explored whether ES-62 affects the osteoimmunology axis that integrates environmental signals, such as diet and the gut microbiome to homeostatically regulate haematopoiesis and training of immune responses, which become dysregulated during (obesity-accelerated) ageing. Of note, we find sexual dimorphisms in the decline in bone health, and associated dysregulation of haematopoiesis and consequent peripheral immune responses, during obesity-accelerated ageing, highlighting the importance of developing sex-specific anti-ageing strategies. Related to this, ES-62 protects trabecular bone structure, maintaining bone marrow (BM) niches that counter the ageing-associated decline in haematopoietic stem cell (HSC) functionality highlighted by a bias towards myeloid lineages, in male but not female, HCD-fed mice. This is evidenced by the ability of ES-62 to suppress the adipocyte and megakaryocyte bias and correspondingly promote increases in B lymphocytes in the BM. Furthermore, the consequent prevention of ageing-associated myeloid/lymphoid skewing is associated with reduced accumulation of inflammatory CD11c^+^ macrophages and IL-1β in adipose tissue, disrupting the perpetuation of inflammation-driven dysregulation of haematopoiesis during obesity-accelerated ageing in male HCD-fed mice. Finally, we report the ability of small drug-like molecule analogues of ES-62 to mimic some of its key actions, particularly in strongly protecting trabecular bone structure, highlighting the translational potential of these studies.

## Introduction

The dramatic increase in lifespan, resulting from advances in medicine, better nutrition and improved sanitation over the last century, has not been mirrored by a similar increase in late-life health and well-being (healthspan). This disconnect reflects that the ageing process appears to have been accelerated by adoption of the Western life-style, incorporating a high calorie diet (HCD) and sedentary behaviours, resulting in dysfunction of immunometabolic networks and promotion of age-associated co-morbidities such as type 2 diabetes (T2D), stroke, cardiovascular disease, and cancers (1). Moreover, there is increasing evidence that HCD-induced obesity (2) acts as a reciprocal risk factor with autoimmune conditions in promoting dysbiosis of the microbiome and disruption of gut barrier integrity, with the resulting inflammation inducing dysregulation of the key cellular sensor that integrates metabolism and inflammation, mTOR (3, 4). This triggers canonical biological ageing processes (1, 5, 6), including the premature ageing of haematopoietic stem cells (HSC) and consequent immune system dysfunction (5, 6). Indeed, we have recently shown that the extended health- and life-span exhibited by mTOR mutant (S6Kinase1-null) mice is associated with preservation of HSC function during ageing (7).

The pivotal role of chronic low-grade inflammation in driving age-associated co-morbidities and (accelerating) ageing *per se* also resonates with the Hygiene Hypothesis in which the evolutionary-rapid eradication of pathogens, including helminths, has left us with unbalanced hyperactive immune systems that may have contributed to the recent alarming increase in prevalence of allergic and autoimmune disorders (8, 9). Consistent with this, evidence from both epidemiological studies and animal models of inflammatory disease suggests that parasitic helminth infection might protect humans from developing chronic inflammatory conditions and indeed, live helminths have undergone trials as therapeutics in a range of immune-mediated diseases (8, 9). Certainly, we have shown that ES-62, a phosphorylcholine (PC)-containing immunomodulator secreted by the filarial nematode, *Acanthoceilonema viteae*, protects against allergic and autoimmune pathology in various mouse models by subverting TLR4-signalling to downregulate aberrant MyD88 responses and restore the regulatory:effector immune system balance, thereby allowing it to homeostatically resolve aberrant inflammation irrespective of its phenotype [reviewed in reference (10)]. Collectively such findings led us to hypothesise that ES-62 might increase health- and lifespan by targeting inflamm-ageing.

We therefore embarked on an age- and sex-matched longitudinal survival study, combined with evaluation of parallel interventional cross-sectional cohorts at various time-points across the life-course that allowed us to analyse effects of ES-62 (when administered at 1 µg/week) on >120 inflammatory and metabolic parameters in C57BL/6J mice fed a HCD diet (11). Male and female mice were found to exhibit distinct pathological responses in this model of obesity-accelerated ageing, with female mice typically showing more exaggerated inflammatory responses whilst the male animals exhibited more pronounced metabolic defects including visceral adipocyte hypertrophy, insulin resistance and loss of pancreatic β-cell function (11). This sexual dimorphism extended to ES-62-responsiveness, with sex-specific improvements in healthspan apparent and strikingly, increasing median lifespan recorded in male, but not female, HCD-fed mice (11). Mathematical modelling identified (as expected) that anti-inflammatory activities were amongst the signatures most predictive of ES-62 action (11). However, this analysis also highlighted the importance of additional protective effects targeting intestinal integrity in male HCD-cohorts. These actions reflected normalisation of the gut microbiome (11) with, in particular, profound depletion of proteobacteria species previously associated with promoting ageing (12, 13). Of note, we have also shown the protection against joint inflammation and bone damage afforded by ES-62, in the collagen-induced arthritis (CIA) mouse model of rheumatoid arthritis, to be associated with its normalisation of the gut microbiome and intestinal barrier integrity (14).

Collectively, these findings may be pertinent to the ability of ES-62 to promote healthspan as gut health impacts not only on the “training” of immune responses and skeletal health (osteoimmunology) (15–17), but also on inflamm-ageing, age-associated comorbidities, frailty and the ageing process *per se* (18–20). This reflects that the combination of cumulative exposure to pathogens and Western-style diets causes changes in the gut microbiota during obesity and ageing that drives the dysregulation of haematopoiesis. Such dysregulation is typically characterised by an increase in HSC numbers but a decline in HSC functionality (HSC exhaustion/senescence) and a bias towards myeloid lineages (21–23). Generation of the resultant inflammatory network termed the senescence-associated secretory phenotype (SASP) impacts on long-term HSC functionality, perpetuating dysregulation of haematopoiesis (24, 25). The SASP is thus manifested by anaemia, immunosenescence and thrombocytosis, as well as elevated systemic levels of cytokines and chemokines (e.g., IL-1α and β, IL-6 and TNFα; CXCL1 and CXCL2) (22, 24). The impact of such decline in HSC functionality is underlined by the evidence that BM Transfer (BMT) from young to old mice can extend lifespan (26, 27), as well as protect against obesity and age-associated comorbidities like cardiovascular and neurodegenerative disease (28, 29). SASP arises due to HSCs responding both directly to microbiota-derived molecules and indirectly, *via* the impairment of the haematopoiesis-supporting BM microenvironment resulting from the impact of inflammation and metabolic stress on mesenchymal lineages (22, 23, 25). As TLR/MyD88 signalling, which is a key target of ES-62, plays an important role in these events, we therefore investigated whether, by modulating bone structure and the BM microenvironment, ES-62 acted to reduce dysregulation of haematopoiesis to suppress inflamm-ageing and promote healthspan during HCD-accelerated ageing of male and female mice.

## Methods

### Ethics statement

All procedures were performed in accordance with UK Home Office Project Licences (60/4504 and PDBDC/7568), following the “principles of laboratory animal care” (NIH Publication No. 86-23, revised 1985) and approval by the University of Glasgow Animal Welfare and Ethical Review Board.

### The obesity-accelerated mouse model

Male and female C57BL/6J mice (Envigo, UK) were housed (in same sex groups of 2 to 4, randomly allocated on arrival at 4 weeks of age) in the Central Research Facility (University of Glasgow, UK) and maintained, under specific pathogen-free conditions, at 22°C under a 12-h light/dark cycle with *ad libitum* access to water and Chow (CRM-P) and High Calorie (Western Diet RD) diets from Special Diet Services, UK as described previously (11, 30). Briefly, from 4 weeks of age, all mice were fed normal Chow CRM-P diet (comprising Oil, 3.36%; Protein 18.35%; Fibre, 4.23%: Sugar 3.9%; Atwater fuel energy from Oil, 9.08%; Protein, 22.03%: Carbohydrate, 68.9%) plus 150 ppm Fenbendazole. Mice were administered PBS, purified endotoxin-free ES-62 (1 μg (31);) or a combination of SMAs 11a plus 12b (both 1 µg (32)) weekly *via* the subcutaneous route from 9 weeks of age (30, 31). At 10 weeks of age, the HCD cohorts received Western Diet RD (Fat, 21.4%; Protein, 17.5%; Fibre, 3.5%; Sucrose 33%; Atwater fuel energy from Fat, 42%; Protein, 15%: Carbohydrate, 43%) plus 150 ppm Fenbendazole.

For the ES-62 time-course study, male and female cohorts of mice were culled at the following ages (days; (d)), d56 (8 weeks); d160 (22-23 weeks); d340 (48-49 weeks) and d500 (71-72 weeks). The group sizes were: d56 (Chow + PBS, n=6), d160 (HCD + PBS/ES-62, n=10/group; Chow + PBS, n=6), d340 (HCD + PBS/ES-62, n=12/group; Chow + PBS, n=6) and d500 (HCD + PBS/ES-62, n=6/group; Chow + PBS, n=5). For analysis of the effect of ES-62 on ageing Chow-fed mice, additional male and female cohorts of mice were culled at 340 days (48-49 weeks) of age (Chow + PBS/ES-62, n=8; HCD + PBS, n=8) and, in the SMA study, male and female cohorts of mice were culled at 160 days (22-23 weeks) of age (Chow + PBS, n=4; HCD + PBS/ES-62, n=6).

Following fasting overnight (~16 h), blood was collected between 8-10 am from mice after cervical dislocation and rapid decapitation of the animals from the severed carotid artery. Blood was either immediately aliquoted into FACS buffer (PBS containing 2.5% BSA and 0.5 mM EDTA) and kept on ice for processing for flow cytometric analysis or following clotting at room temperature, centrifuged and the isolated serum stored at-20°C in endotoxin-free Eppendorf tubes for analysis of IL-1β and TNF-α by ELISA (BD Biosciences, Oxford UK).

### Bone histology and micro computed tomography (µCT)

Paws and femurs were fixed in 4% paraformaldehyde prior to decalcification and paraffin wax embedding for H&E staining (7 μm sections) of joints as described previously (14, 33). Joint histopathology was determined by semi-quantitative scoring of cell infiltration/pannus formation, cartilage and bone erosion, each scored on a 0-3 severity scale (34) and then averaged, with the mean values representing the histopathology scores for individual mice. In bone sections, adipocytes were identified visually in the BM by their spherical, amorphous and white appearance. BM adipocytes were enumerated in 3 images from each mouse and the numbers of adipocytes normalised to the area of the BM, calculated using ImageJ software (pixel to micron conversion) for each field of view, with the mean values calculated as the data point for each individual animal.

Femurs were subjected to µCT analysis at the Microscopy and Histology Core Facility at the Institute of Medical Sciences, University of Aberdeen (https://www.abdn.ac.uk/ims/facilities/ microscopy-histology/services-and-equipment-1872.php#panel1876), using a Skyscan1072 x-ray Microtomograph (Skyscan, Aartselaar, Belgium) and NRecon version 1.4.4. (3D images) and CTan version 1.0.7.2 Skyscan software using protocols described previously (35–37).

### Flow cytometry

Flow cytometric analysis was performed as described previously (11, 14, 30, 33). Briefly, following red cell-lysis (with 0.8% NH_4_Cl buffer), BM, blood, spleen, MLN and adipose tissue cells were suspended in FACS buffer (PBS containing 2.5% BSA and 0.5 mM EDTA) and phenotyped using the following antibodies:

**Table:** 

Tissue	Specificity	Conjugate	Clone	Supplier	Catalogue #
BM	CD3	PE	17A2	BioLegend	#100205
	B220	PE	RA36B2	BioLegend	#103207
	Ter119	PE	TER-119	BioLegend	#116207
	CD11b	FITC	M1/70	BioLegend	#101206
	Sca-1	FITC	D7	BioLegend	#108116
	Ly6C	PerCP-Cy5.5	HK1.4	BioLegend	#128011
	Ly6G	APC	1A8	BioLegend	#127613
	CD11b	APC	M1/70	BioLegend	#101212
	CD45	APC	30-F11	BioLegend	#103112
	RANKL	Biotin	IK22/5	BioLegend	#510003
	CD117	Biotin	2B6	BioLegend	#105803
	Streptavidin	APC-Cy7		BioLegend	#405208
	CD115	Biotin	AFS98	eBioscience	#13-1152-82
	CD117	APC	2B8	eBioscience	#17-1171-82
Other tissues	CD3	FITC	146-2C11	BioLegend	#100305/6
	CD3	FITC	17A2	BioLegend	#100203
	CD3	PE	17A2	ImmunoTools	#22150034
	CD4	PE	GK1.5	BioLegend	#100407
	CD4	APC-ef780	RM4-5	eBioscience	#47-0042-82
	CD8	PE-Cy7	53-6.7	eBioscience	#25-0081-82
	CD44	PerCP	IM7	BioLegend	#103036
	CD45RB	FITC	C363-16A	BioLegend	#103305
	CD45RB	APC-ef780	30-F11	Invitrogen	#47-0451-82
	CD19	AF700	6D5	BioLegend	#115527/8
	CD11c	FITC	N418	BioLegend	#117305
	CD11c	PE-Cy7	N418	BioLegend	#117318

HSC analysis involved use of a dump channel (PE-conjugated lineage cocktail) in combination with FITC anti-Sca-1 and APC or biotin anti-CD117 antibodies. For surface marker staining, antibodies were used at 0.2 µg/10^6^ cells (1/100 dilution) except for anti-CD45 (1/200 dilution). Streptavidin was used at 1/500 dilution. Cell death was assessed by fixed viability stain (APC-ef780) or 7AAD (BD Bioscience, UK) staining. Data were acquired using FACS Canto or BD LSRII flow cytometers and analysed using FlowJo Software (Tree Star In, OR USA, version 8.8.7) and populations were gated using isotype and fluorescence minus one (FMO) controls (11, 14, 30, 33). Exemplar gating strategies are shown in **Supplementary Figure 1**.

### Osteoclast (OC) differentiation *in vitro*

OC differentiation was determined as we described previously (14, 33). Briefly, BM was flushed from the tibias and femurs using a sterile 23-gauge needle and syringe and aspirated to create a single cell suspension in PBS which, following filtering through a 20 µm cell strainer, was centrifuged (400 x g), washed in PBS and resuspended (10^6^/ml) in “complete” αMEM medium (containing 50 U/ml penicillin, 50 μg/ml streptomycin and 10% FCS). Cells were incubated overnight with M-CSF (30 ng/ml; Peprotech, London, UK) at 37°C in 5% CO_2_ and then following removal of non-adherent cells, re-suspended in fresh complete αMEM medium supplemented with 30 ng/ml M-CSF and 50 ng/ml RANKL and OC differentiation initiated by seeding cells (10^5^) in 96-well tissue-culture plates, with the medium refreshed on day 4. Functional maturation was assessed by TRAP staining (Leukocyte Acid Phosphatase Kit, Sigma, UK) on day 5, with TRAP^+^ cells containing ≥ 3 nuclei counted as OCs. The size of individual OCs per field of view (FOV) was calculated from images (x4 magnification; scale bars 1000 µm) generated by an EVOS FL Auto Cell Imaging System using Image J software.

### qRT-PCR

As described previously (14, 33), BM cells (10^6^) were lysed in RNeasy Lysis Buffer prior to mRNA extraction using RNeasy Plus Mini kit (Qiagen, Germany) and cDNA generated using the High Capacity cDNA Reverse Transcriptase kit (Applied Biosystems, Life Technology) and amplified using the StepOne Plus™ real-time PCR system (Applied Biosystems). KiCqStart^®^ qPCR Ready Mix (Sigma-Aldrich) was used in conjunction with the following primer pairs:

**Table:** 

Gene	Forward Primer	Reverse Primer
RANK, *tnfrsf11a*	GAAATAAGGAGTCCTCAGGG	TAGAATCTCTGACTTCTGCC
RANKL, *tnfsf11*	TCTGTTCCTGTACTTTCGAG	TTCATGGAGTCTCAGGATTC
OPG, *tnfrsf11b*	GAAGATCATCCAAGACATTGAC	TCCTCCATAAACTGAGTAGC
IL-1β *il1b*	GTGATATTCTCCATGAGCTTTG	TCTTCTTTGGGTATTGCTTG
β-actin, *actb*	GATGTATG AAGGCTTTGGTC	TGTGCACTTTTATTGGTCTC

Data were normalised to the reference gene β-actin to obtain the ΔCT values and expressed as Rq (2^-ΔCt).

### Statistical analysis

All data were analysed by GraphPad Prism 9 software using unpaired student T-tests, one or two-way ANOVA with Fishers LSD post-test for parametric data or Kruskal-Wallis test for non-parametric/ordinal data. The data presented in scatter plots are the mean values (of triplicate images/assays) or, for flow cytometric analysis, the % live cells or Mean Fluorescence Intensity (MFI) values of the individual mice in the group (bar is the mean value for the group) and analysed by one-way ANOVA. For time-courses, the data are presented as the mean ± SEM values for the group (n values presented in legends) derived from the mean values for the individual mice and analysed by two-way ANOVA. Significant differences between the cohorts are shown on the figures, where significance is denoted by ^*^p < 0.05, ^**^p < 0.01, ^***^p < 0.001 and ^****^p<0.0001 or ^#^p < 0.05, ^##^p < 0.01, ^###^p < 0.001 and ^####^p<0.0001.

## Results

**Figure 1:**
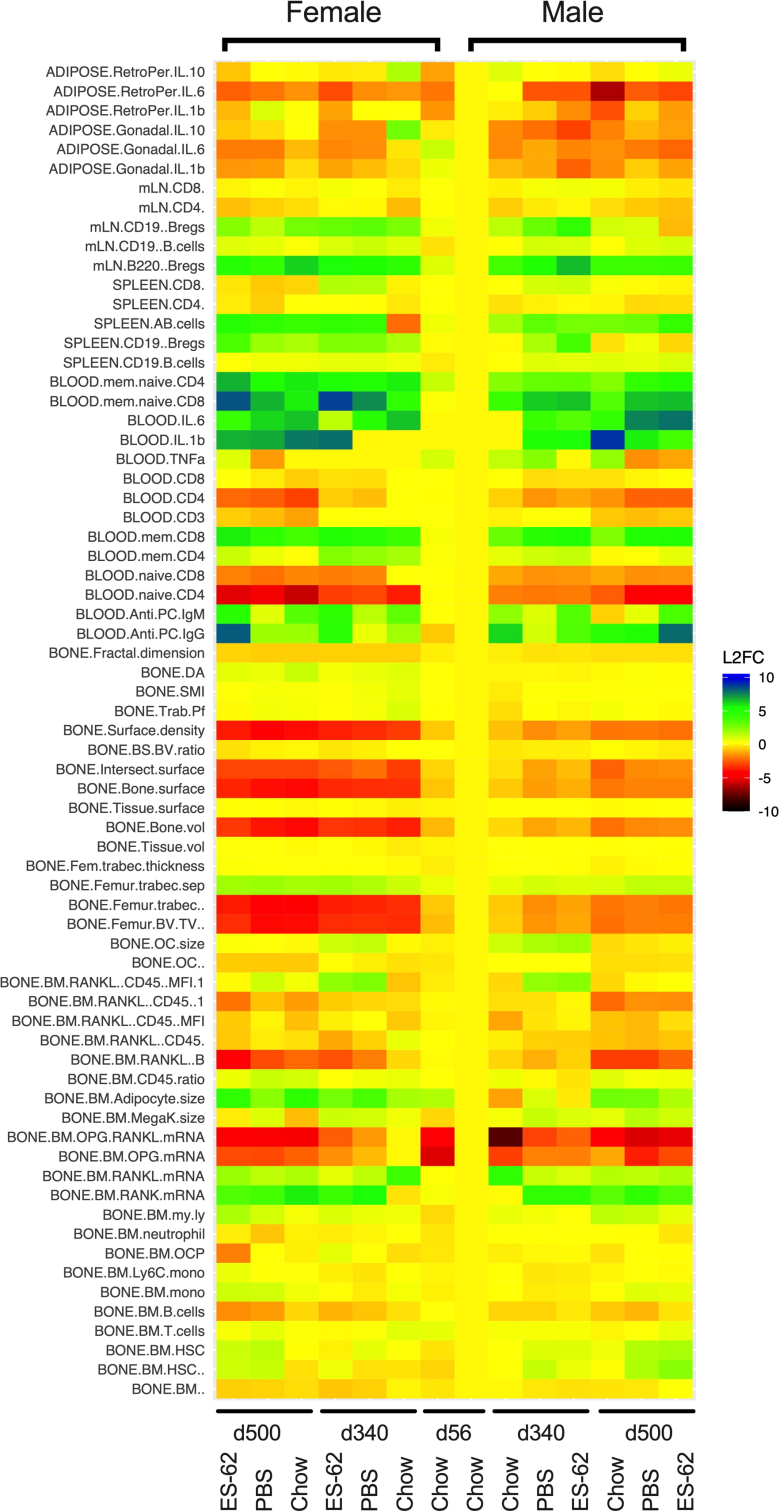
Heatmap analysis of the effects of HCD-feeding and exposure to ES-62 on parameters associated with the osteoimmunology axis. Characterisation of the indicated features of bone structure, bone marrow cellularity, differentiation and myeloid/adipocyte skewing and consequent systemic and peripheral inflammatory responses was carried out on tissues derived from individual young (d56) and ageing (d340 and d500) male and female mice fed normal Chow or a HCD (from d70) diet, the HCD groups being administered (s/c) PBS or ES-62 (1 µg) weekly from d63. The mean responses of the groups were determined and normalised to those of the male d56 group and the data presented in heatmap form in terms of the log2 fold response for each parameter.

**Figure 2:**
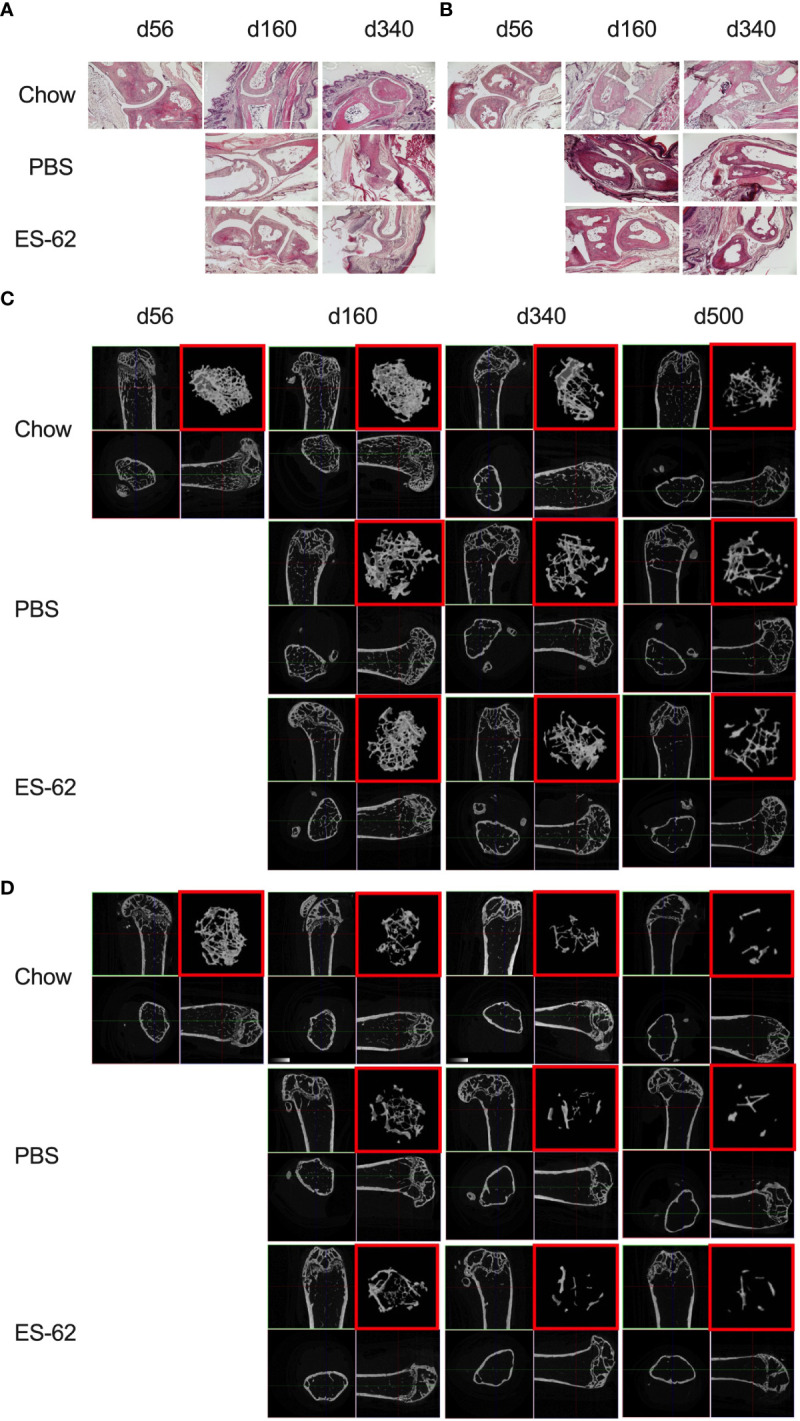
Impact of HCD-feeding and exposure to ES-62 on trabecular bone structure. Representative sections of H & E staining of paw joint sections (scale bar 400 µm) are shown for the indicated groups of male **(A)** and female **(B)** mice and where tissue damage was not significantly different across the groups. Scores: for male mice, d56: 0, n=3, Chow: 0.48 ± 0.09, n=11; PBS-HCD: 0.58 ± 0.08, n=4; ES-62: 0.25 ± 0.08, n=4; for female mice, d56: 0.11 ± 0.11, n=3; Chow: 0.50 ± 0.14, n=10; PBS-HCD: 0.66 ± 0.24, n=4; ES-62: 0.44 ± 0.22, n=3. MicroCT analysis of femurs from the indicated groups of male **(C)** and female **(D)** mice was performed and representative 2D images of transverse sections through femurs illustrating the reference growth plate and the downstream area (~200 slices of pixel size 5 µm) of analysis of trabecular parameters (upper left and lower right boxes) are shown. Reconstruction of the image stack provided 3D images of the trabecular structure (red box) whilst cross-sectional analysis provides a 2D image of the cortical bone (lower left box).

**Figure 3:**
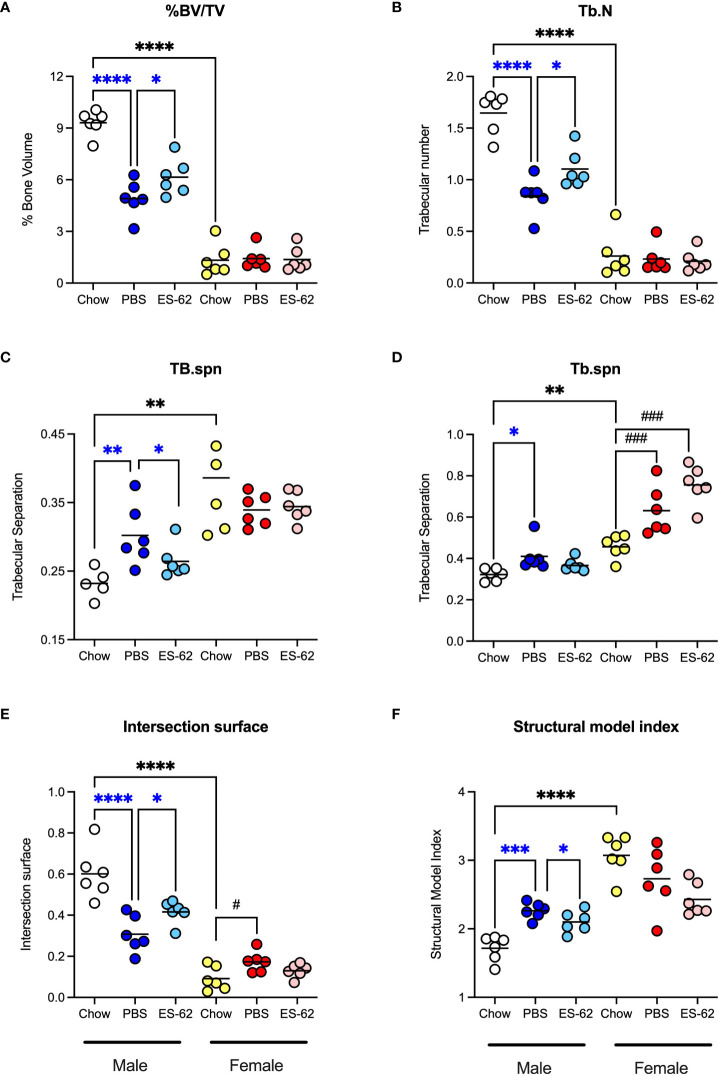
ES-62 protects against the HCD-accelerated changes in parameters of trabecular bone architecture in male mice. The effects of ES-62 on HCD (PBS)- and ageing-induced decline in bone structure homeostasis as evidenced by changes in **(A)** % Bone Volume/Tissue Volume (BV/TV) at d340; **(B)** Trabecular Number (Tb.N) at d340; **(C)** Trabecular Separation (Tb.spn) at d160; **(D)** Trabecular Separation (Tb.spn) at d340; **(E)** Intersection Surface at d340 and **(F)** Structure Model Index (SMI) at d340. The data shown are the values of femurs from individual mice (symbols) with the mean value for the group represented by the bar and significant differences indicated by blue****=p<0.0001 for male PBS v male Chow, blue***= p<0.001 for male PBS v male Chow, blue**=p<0.01 for PBS v male Chow, blue*=p<0.05 for male PBS v male Chow and blue*= p<0.05 for male PBS v male ES-62 **(A–F)**, black****= p<0.0001 for male Chow v female Chow and black**=p<0.01 for male Chow v female Chow **(A–F)**, black^###^ = p<0.001 for female Chow v female PBS or female ES-62 **(D)** and black^#^ = p<0.05 for female Chow v female PBS **(E)** groups.

**Figure 4:**
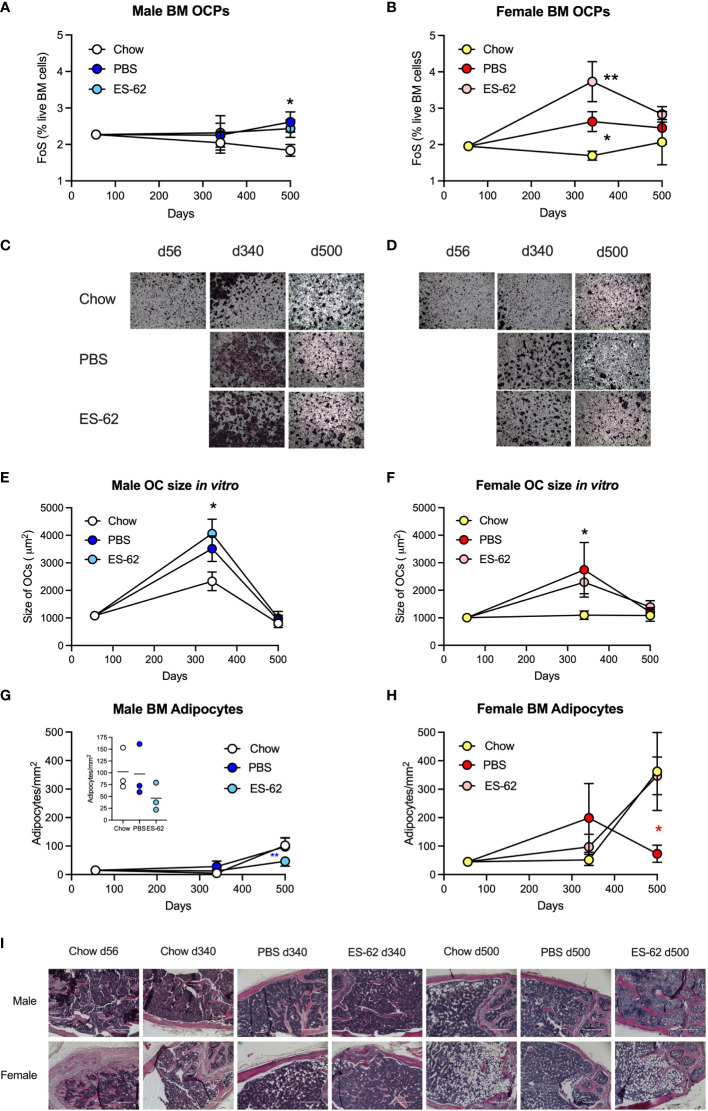
Obesity-accelerated ageing is associated with enhanced osteoclastogenic potential and BM adipocyte accumulation. The levels of BM CD3^−^B220^−^Ter119^−^Ly6G^−^Ly6C^high^ CD11b^low^ OCPs were determined as the frequency of single cells (FoS) expressed as % live BM cells at the indicated timepoints in **(A)** male and **(B)** female mice fed Chow or HCD, the latter cohort treated with either PBS or ES-62. The data are presented as the mean ± SEM values for the indicated group, where for male mice, at d56: n=6; d340: Chow, n=6; PBS, n=7; ES-62, n=8; d500, Chow, n=5; PBS, n=5; ES-62, n=5 individual mice and for female mice, at d56: n=6; d340: Chow, n=6; PBS, n=8; ES-62, n=8; d500, Chow, n= 3; PBS, n=5; ES-62, n=6 individual mice. Significant differences are indicated by *=p<0.05, male Chow v male PBS or ES-62 **(A)** and female Chow v female PBS **(B)** and **=p<0.01, female Chow v female ES-62 **(B)** groups. **(C–F)** OC differentiation at day 5 was assessed by TRAP staining and the size of multinucleated OCs (>3 nuclei) determined by Image J analysis. Representative images (x4 magnification) of OCs of the indicated groups of male **(C)** and female **(D)** mice are shown and the mean group OC size ± SEM determined from the mean values of triplicate cultures of individual male mice at each time point where for male mice **(E)**, at d56: n=6; d340: Chow, n=6; PBS, n=3; ES-62, n=4; d500, Chow, n=5; PBS, n=5; ES-62, n=5 individual mice and for female mice **(F)**, at d56: n=6; d340: Chow, n=6; PBS, n=3; ES-62, n=4; d500, Chow, n=4, PBS, n=5; ES-62, n=5 individual mice. Significant differences are indicated by *=p<0.05, Chow v PBS or ES-62 for male **(E)** and female **(F)** mice. **(G–I)** BM adipocytes were visualised (x10 magnification, scale bars 400 µm) and quantitated by Image J analysis where the mean group number/mm^2^ ± SEM is determined from the mean values of triplicate fields of view (FoV) of the individual mice at each time point. For male mice **(G)**, d500 data scale expanded in insert), d56: n=6; d340: Chow, n=6; PBS, n=6; ES-62, n=6; d500, Chow, n=3; PBS, n=3; ES-62, n=3 individual mice and for female mice **(H)**, d56: n=6; d340: Chow, n=5, PBS, n=5; ES-62, n=6; d500, Chow, n=3; PBS, n=3; ES-62, n=3 individual mice. Significant differences are indicated by blue**=p<0.01 for male PBS v male ES-62 **(G)** and red*=p<0.05, female PBS v female ES-62 **(H)** groups. Representative images are shown for each group **(I)**.

**Figure 5:**
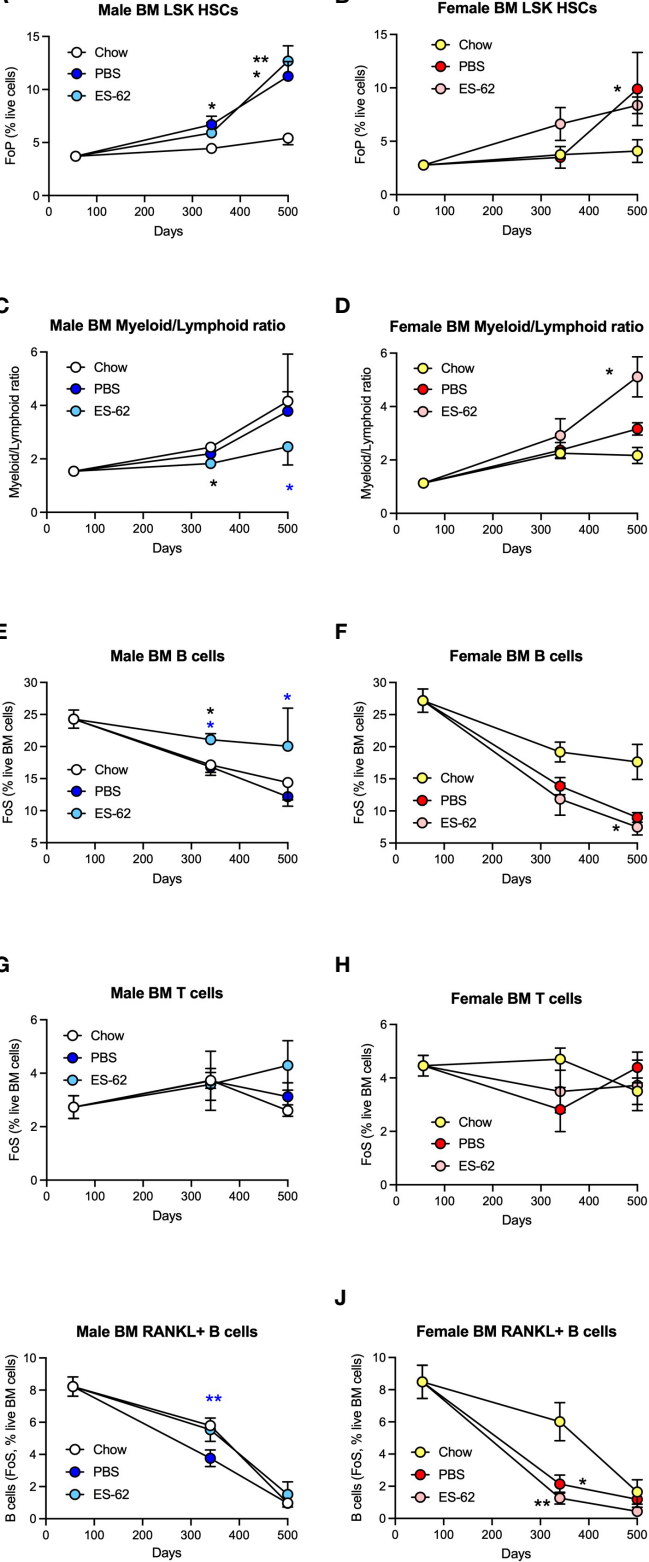
ES-62 protects against myeloid/lymphoid skewing and maintains B cell levels in the BM of male HCD-fed mice. The levels of Lin^-^Sca-1^+^c-kit^+^ (LSK) HSC (**A**, male; **B**, female; as % Lin^-^ cells [frequency of precursor (FoP)]) and the ratios of Myeloid/Lymphoid lineages (**C**, male; **D**, female) are shown. The levels of CD19^+^ B cells **(E, F)**, CD3^+^ T cells **(G, H)** and RANKL^+^ B cells **(I, J)** were determined as the frequency of single cells (FoS) expressed as % live BM cells at the indicated timepoints in male **(E, G, I)** and female **(F, H, J)** mice fed Chow or HCD, the latter cohort treated with either PBS or ES-62. The data are presented as the mean ± SEM values for individual mice in each group, where for male mice, at d56: n=6; d340: Chow, n=6; PBS, n=7; ES-62, n=8; and at d500, Chow, n=5; PBS, n=5; ES-62, n=5 and for female mice, at d56: n=6; d340: Chow, n=6; PBS, n=8; ES-62, n=8; and at d500, Chow, n= 3; PBS, n=5; ES-62, n=6. Significant differences are indicated by black*=p<0.05 for Chow v PBS or ES-62 in male **(A, C, E)** or female **(B, D, F, J)** mice; black**=p<0.01 for Chow v PBS or ES-62 in male **(A)** and female **(J)** mice; blue*=p<0.05 for male PBS v male ES-62 **(C, E)** and blue **=p<0.01 for male PBS v male ES-62 **(I)** groups.

**Figure 6:**
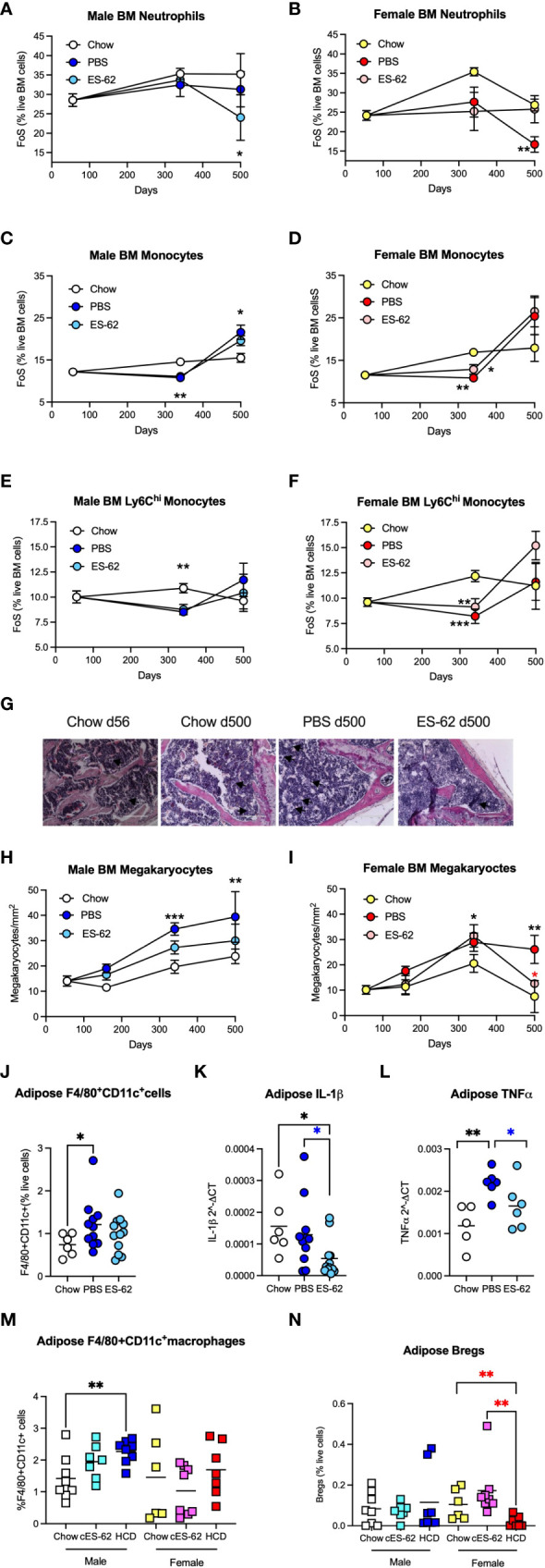
ES-62 modulates levels of BM myeloid lineages in male HCD-fed mice. The levels of Ly6C^+^Ly6G^+^ neutrophils **(A, B)**, Ly6C^+^Ly6G^-^ monocytes **(C, D)** and Ly6C^high^ monocytes **(E, F)** were determined as the frequency of single cells (FoS) expressed as % live BM cells at the indicated timepoints in male **(A, C, E)** and female **(B, D, F)** mice fed Chow or HCD, the latter cohort treated with either PBS or ES-62. The data are presented as the mean ± SEM values for individual mice in each group, where for male mice, at d56: n=6; d340: Chow, n=6; PBS, n=7; ES-62, n=8 and at d500, Chow, n=5; PBS, n=5; ES-62, n=5 and for female mice, at d56: n=6; d340: Chow, n=6; PBS, n=8; ES-62, n=8 and at d500, Chow, n= 3, PBS, n=5; ES-62, n=6. Significant differences are indicated by black*=p<0.05 for Chow v PBS or ES-62 in male **(A, C)** or female **(D)** mice; black**=p<0.01 for Chow v PBS or ES-62 in male **(C, E)** and female **(B, D, F)** mice and black***=p<0.001 for Chow v PBS in female mice **(F)** groups. **(G–I)** BM megakaryocytes were visualised (x20 magnification, scale bars 200 µm), with representative images shown for the indicated groups of male mice **(G)**. Quantitation was by Image J analysis where the mean group number/mm^2^ ± SEM is determined from the mean values of triplicate field of view (FoV) of individual male **(H)** and female **(I)** mice at each time point. For male mice, at d56: n=6; d340: Chow, n=6; PBS, n=6; ES-62, n=6; and at d500, Chow, n=3; PBS, n=3; ES-62, n=3 and for female mice, at d56: n=6; d340: Chow, n=5; PBS, n=5; ES-62, n=6 and at d500, Chow, n=3; PBS, n=3; ES-62, n=3. Significant differences are indicated by black**=p<0.01 and black***=p<0.001 for male Chow v male PBS but not male ES-62 **(H)** and black*=p<0.05 for female Chow v female PBS or female ES-62; black**=p<0.01 for female Chow v female PBS and red*=p<0.05 for female PBS v female ES-62 **(I)** groups. Levels of F4/80^+^CD11c^+^ macrophages **(J, M)**, IL-1β **(K)** and TNFα **(L)** mRNA and IL-10^+^CD19^+^ Bregs **(N)** were determined in gonadal adipose tissue of each of Chow-, HCD-PBS and HCD-ES-62 male mice **(J–L)** and in separate d340 studies investigating the effect of ES-62 in a non-obese setting involving Chow-, Chow-ES-62 (cES-62) and, as a confirmatory obesity-accelerated ageing control, HCD-PBS male and female mice cohorts **(M, N).** The data shown are mean values for individual mice (symbols) and the group means are indicated by the bars. Significant differences are indicated by black*=p < 0.05 for male Chow v male PBS **(J)** or ES-62 **(K)**; black**=p<0.01 for male Chow v PBS **(L)**; blue*=p<0.05 for male PBS v male ES-62 **(K, L)**; black**=p<0.01 for male Chow v HCD **(M)** and red**=p<0.01 for female HCD v female Chow and female cES-62 **(N)** groups.

**Figure 7:**
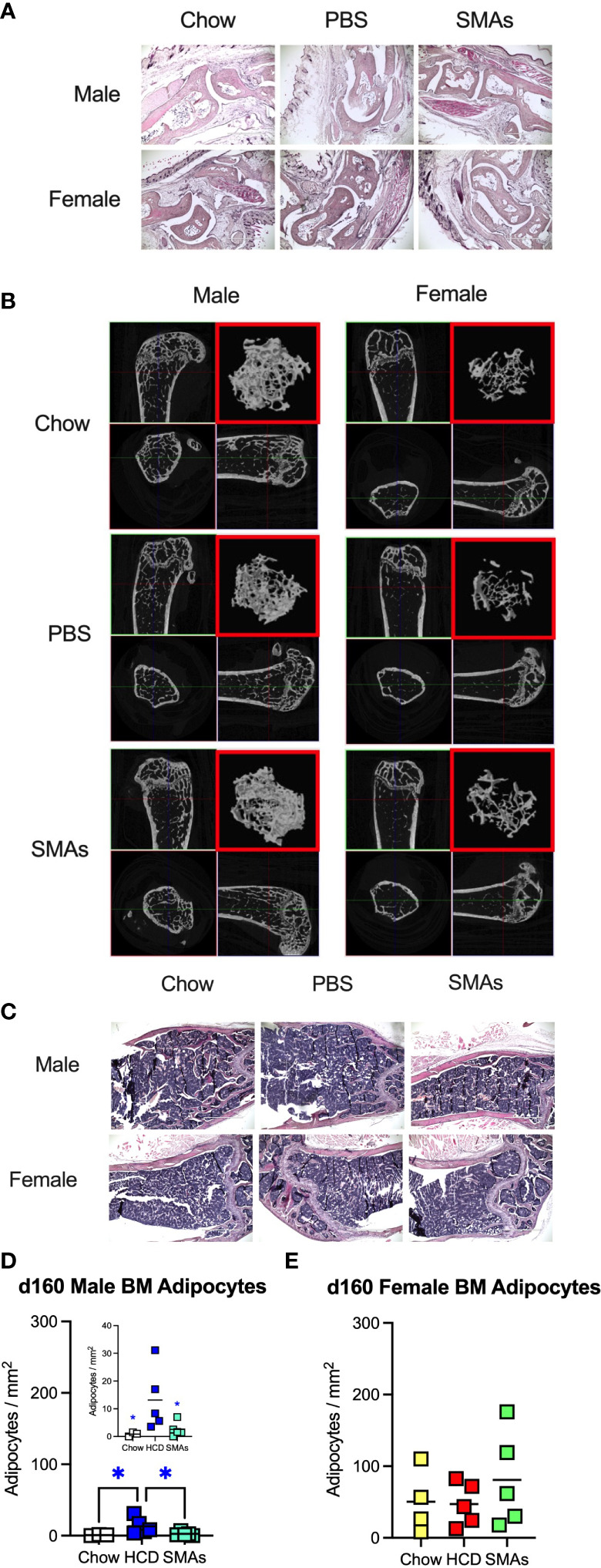
ES-62-based SMAs protect against disruption of the BM niche in male HCD-fed mice at d160, a time-point when substantial (obesity-accelerated) loss of trabecular bone structure is established. **(A)** Representative sections of H & E staining of paw joint sections (scale bar 1000 µm) are shown for the indicated groups of d160 male and female mice and where tissue damage was not significantly different across the groups. Scores: for male mice, Chow: 0.66 ± 0.19, n=4; PBS-HCD: 0.38 ± 0.10, n=6; SMAs-HCD: 0.50 ± 0.17, n=6; for female mice, Chow: 0.58 ± 0.16, n=4; PBS-HCD: 0.53 ± 0.17, n=5; SMAs-HCD: 0.33 ± 0.15, n=5. **(B)** MicroCT analysis of femurs from the indicated groups of d160 male and female mice was performed and representative 2D images of transverse sections through femurs illustrating the reference growth plate and the downstream area (~200 slices of pixel size 5 µm) of analysis of trabecular parameters (upper left and lower right boxes) are shown. Reconstruction of the image stack provided 3D images of the trabecular structure (red box) whilst cross-sectional analysis provides a 2D image of the cortical bone (lower left box). **(C)** Representative images of BM adipocytes (x10 magnification, scale bars 400 µm) in male and female Chow-, HCD-PBS- and HCD-SMAs-fed mice at d160 are shown. **(D, E)** Quantitation is by Image J analysis where the mean group number/mm^2^ ± SEM is determined from the mean values (of triplicate FoV values) of the individual mice (symbols) and the group means are indicated by the bars for male (D; insert shows expanded scale) and female **(E)** mice. Significant differences are indicated by blue*=p < 0.05 for male HCD-PBS v male Chow or male HCD-SMAs mice **(D)**.

**Figure 8:**
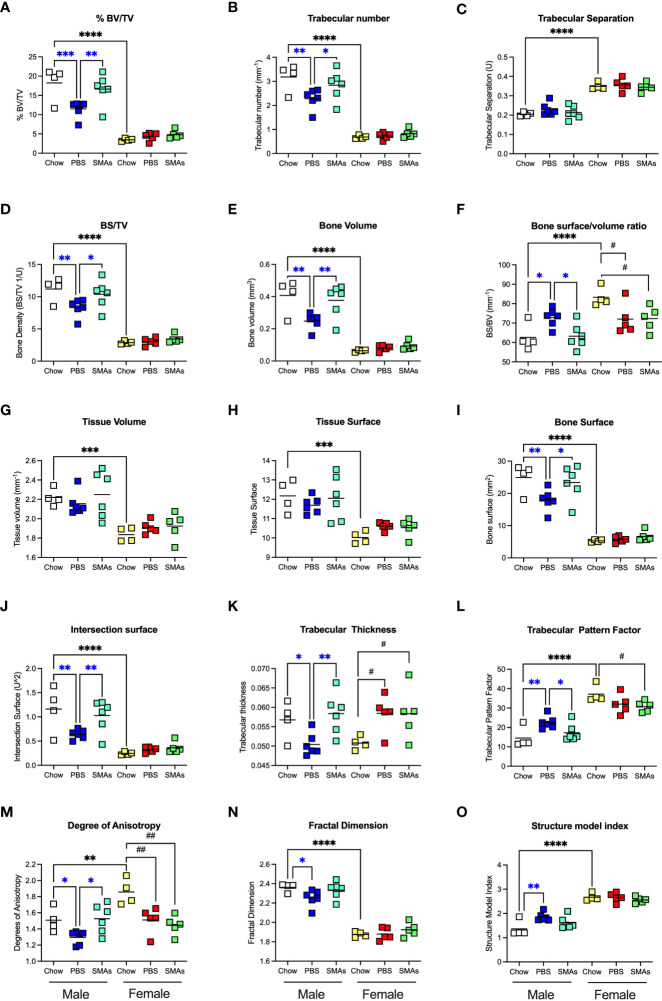
ES-62-based SMAs protect against the HCD-accelerated decline in trabecular bone structure in male HCD-fed mice. The effects of ES-62-based SMAs on the HCD (PBS)- accelerated decline in bone structure homeostasis, as evidenced by changes in the indicated parameters of trabecular architecture in femurs from Chow-, HCD-PBS (PBS)- and HCD-SMAs (SMAs) groups of male and female mice **(A–O)** at d160. The data shown are the values of femurs from individual mice (symbols) with the mean value for the group represented by the bar. Significant differences are indicated by blue***=p<0.001 for male PBS v male Chow, blue**=p<0.01 for male PBS v male Chow or male SMAs, and blue*=p<0.05 for male PBS v male Chow or male SMAs **(A, B, D–F, I–O)**; black****=p<0.0001, black***=p<0.001 and black**=p<0.01 for male Chow v female Chow **(A–J, L–O)** and black^##^=p<0.01 and black^#^=p<0.05 for female Chow v female PBS or female SMAs **(F, K–M)** groups.

We hypothesised that ES-62 acts to normalise the osteoimmunology axis in its suppression of inflamm-ageing and promotion of healthspan during obesity-accelerated ageing. Thus, we analysed the impact of HCD-feeding on ~70 parameters of osteoimmunology - ranging through bone structure and pathology to BM cellularity, differentiation, lineage skewing and consequent peripheral immune responses – across the lifecourse in male and female C57BL/6J mice and determined whether ES-62 could modulate any altered responses back towards those exhibited by Chow-fed controls. The data obtained exhibited, sex-, diet- and age-associated signatures that displayed sexual dimorphism with respect to ES-62 responsiveness (summarised in **Figure 1**). The key findings are detailed below (**Figures 2**–**8**), with the remainder of the data provided in the Supplementary Information (**Supplementary Figures 2-13**).

### ES-62 protects against ageing-induced loss of bone structure in male HCD-fed mice

Bone remodelling, balancing the actions of osteoclasts (OCs) and osteoblasts (OBs) in controlling bone resorption and synthesis respectively, is a normal and necessary physiological process during adulthood, but one which becomes dysregulated during obesity and ageing, resulting in loss of bone mass and development of osteolytic diseases such as arthritis and osteoporosis (38–40). In our study, histological analysis of paw synovial joints indicated a trend for limited damage to develop between d160 and d340, but there were no statistically significant differences amongst any of the groups in either sex over this time period (**Figures 2A, B**).

Obesity and ageing have also been reported to impact on trabecular bone architecture with consequent perturbation of the bone marrow niche supporting stem cell function (25), resulting in the skewing of immune responses and development of inflamm-ageing/SASP, with in turn, the further fostering of osteolytic disease and frailty (38–40). Imaging by micro-computed tomography (µCT) revealed clear ageing-associated loss of trabecular bone structure (as indicated by transverse 2D and deconvolved 3D images), in the femurs of Chow-fed male and particularly female mice (**Figures 2C, D** respectively). This process was substantially accelerated by HCD-feeding in male mice whereas the female mice showed profound changes by d160 even when fed a normal Chow diet, confirming the early onset and profound deterioration in trabecular architecture previously reported for this sex of C57BL/6 mouse (41). By contrast, and perhaps consistent with the marginal articular pathology observed (**Figures 2A, B**), changes in cortical (as evidenced by cross-sectional 2D images) bone were much less pronounced, with no dramatic effects observed amongst the groups in either sex at least over the 500 days monitored (**Figures 2C, D**). Quantitative µCT analysis of key parameters of bone health (**Supplementary Figures 2A-O**; **Table 1**) showed that those associated with bone integrity, mechanical function and strength, such as high bone/tissue volume ratios (%BV/TV), bone density (BS/TV), trabecular number (Tb.N), bone intersection surface (contact with trabecular nodes) and fractal dimension, declined during (obesity-accelerated) ageing of the mice. By contrast, the scores for parameters positively associated with osteopenia and osteoporosis (42–44) such as Degrees of Anistropy, Structural Model Index (SMI; plate to rod transition) and trabecular thickness (Tb.Th), separation (Tb.Spn) and pattern (Tb.PF; disconnected trabecular lattice) increased. Noteworthy, loss of bone structure was evident in both sexes of Chow- and HCD-fed ageing mice: however, female mice exhibited a significantly more profound decline than their male counterparts such that their loss of bone structure was not accelerated by HCD administration (**Supplementary Figures 2A-O**). ES-62 was able to significantly reduce a number of the obesity-accelerated changes in trabecular architecture (**Figures 2C, D**; **Figures 3A–F**; **Table 1**) in the male HCD-fed mice, delaying the decline in %BV/TV, Tb.N and intersection surface (**Figures 3A, B, E**) and the increase in trabecular separation and SMI values (**Figures 3C, D, F**). However, perhaps reflecting the deterioration associated with these parameters noted even in the Chow-fed female group, there was no evidence that ES-62 exerted such protective actions in HCD-fed mice of this sex (**Figures 3A–F**; **Table 1**).

**Table 1:** μct analysis of femur bone structure during HCD accelerated in ageing male and female C57BL/6J mice.

	Male									Female									Male v Female Chow		Male Chow v HCD	Female Chow v HCD	Male v Female HCD
	Chow Mean±SEM			HCD Mean±SEM			HCD-ES-62 Mean±SEM			Chow Mean±SEM			HCD Mean±SEM			HCD-ES-62 Mean±SEM			d56	Age-related change (d56-500)			
	d56	d340	%d56^*^	d340	%d56^*^	%d340^†^	d340	%d56^*^	%d340^†^	d56	d340	%d56^*^	d340	%d56^*^	%d340^†^	d340	%d56^*^	%d340^†^	p^‡^	p^§^	p^§^	p^§^	p^§^
BV/TV (%)	14.48 ± 0.99	9.30 ± 0.30	64	4.91 ± 0.43	34	53	6.15 ± 0.43	42	66	7.72 ± 0.65	1.33 ± 0.38	17	1.42 ± 0.26	18	107	1.37 ± 0.70	18	103	<0.0001	<0.0001	0.0004	ns	<0.0001
Tb.N (mm-¹)	2.74 ± 0.13	1.64 ± 0.08	60	0.84 ± 0.07	31	51	1.10 ± 0.07	40	67	1.63 ± 0.14	0.26 ± 0.09	16	0.23 ± 0.05	14	89	0.21 ± 0.04	13	81	<0.0001	<0.0001	<0.0001	ns	<0.0001
Tb.spn (μm)	202 ± 0.34	322 ± 1.21	159	410 ± 2.94	203	128	366 ± 2.99	181	114	307 ± 2.42	457 ± 2.28	149	632 ± 4.79	206	138	756 ± 3.80	246	166	0.0008	<0.0001	0.0004	0.0066	<0.0001
IS	0.77 ± 0.04	0.60 ± 0.05	78	0.31 ± 0.04	40	52	0.42 ± 0.02	54	69	0.54 ± 0.03	0.09 ± 0.02	17	0.17 ± 0.02	32	189	0.13 ± 0.01	24	142	0.0008	<0.0001	0.0001	ns	<0.0001
SMI	1.92 ± 0.06	1.72 ± 0.08	90	2.26 ± 0.05	118	132	2.10 ± 0.06	110	122	2.14 ± 0.5	3.07 ± 0.12	144	2.73 ± 0.19	128	88	2.43 0.10	113	79	0.0057	<0.0001	0.0081	ns	<0.0001

*Treatment value at d340 as % of day d56 value, calculated by regression of mean values.

†Treatment value at d340 as % of d340 Chow control.

‡p value as assessed by Sudent’s t-test.

§p value of sex-by age interaction across time course (d56-500) as determined by 2-way ANOVA.

BV/TV, bone volume/tissue volume; Tb. N, trabecular number; Tb.spn, trabecular bone separation; IS, intersection surface; SMI, structural model index.

To determine whether the observed bone damaging changes were associated with dysregulation of bone remodelling towards osteoclastogenesis, we first examined the levels of BM osteoclast progenitors (OCPs; CD3^−^B220^−^Ter119^−^Ly6G^−^Ly6C^high^ CD11b^low^ BM cells (14, 33). This revealed that although OCP levels remained relatively constant in Chow-fed mice of both sexes up to 500 days of age, they were significantly increased in female, and to a lesser extent in male, HCD-fed mice. However, this HCD-induced increase was not significantly reduced by exposure to ES-62 in either case (**Figures 4A, B**). Rather, we observed a stronger upregulation of OCP levels driven by ES-62 in the BM of HCD-fed female mice (**Figures 4A, B**). Whilst the HCD-induced increase in OCP levels was confirmed in additional cohorts of female mice (**Supplementary Figure 3A, B**), the ES-62 effect was not recapitulated in the “normal” Chow-fed (cES-62) ageing context (**Supplementary Figure 3A, B**). Next, analysis of *in vitro* differentiation of BM OCPs to large, active multinucleated OCs (14, 33), showed that OCPs from both sexes of HCD-fed mice exhibited a greater increase in osteoclastogenic potential at d340 than their Chow-fed counterparts. Again, these HCD-induced changes in OCP functionality were not modulated by *in vivo* exposure to ES-62, but in all groups, declined towards or below the levels exhibited by young mice at d500 (**Figures 4C–F**; **Supplementary Figure 3C, D**). Further evidence that the parasitic worm product was not modulating osteoclastogenesis was provided by the findings that whilst the expression of the OC-promoting factor RANKL (cell surface intensity; MFI) and its receptor, RANK (mRNA) were similarly increased at d340 in BM cells from both sexes of HCD-fed mice, relative to their Chow-fed controls, again, these responses were generally not modulated by ES-62 (**Supplementary Figure 3E-J**).

Collectively these data suggested that the ES-62-mediated protection against the accelerated decline in bone health observed in male HCD-fed mice did not reflect modulation of OCP levels or osteoclastogenesis. Interestingly therefore, and presumably reflecting the mesenchymal stem cell (MSC) impairment that results in an ageing-associated BM adipocyte bias (at the expense of osteoblasts) (45), adipocyte numbers increased in both male and particularly, female mice as they aged (**Figures 4G–I**). Exposure to ES-62 reduced adipocyte numbers in male HCD-fed mice (**Figures 4G, I**), suggesting it may support the osteoblastic niche in mediating its protective effects. Rather surprisingly however, whilst BM adipocyte numbers peaked at d340 in the female HCD-fed mice, the highest numbers of BM adipocytes were found in Chow- and ES-62-HCD-fed female mice at d500 (**Figures 4H, I**). Clearly therefore, sexual dimorphism exists in ageing-associated adipogenesis in the BM.

### ES-62 normalises the ageing-associated myeloid/lymphoid bias of male HCD-fed mice

As the bone stromal microenvironment impacts on HSC functionality, we next investigated how HCD-feeding and exposure to ES-62 impacted on haematopoiesis and subsequent immune responses. Whilst it has been widely reported that the levels of Lin^-^Sca-1^+^c-Kit^+^ (LSK) HSCs are dramatically increased in “old” C57BL/6 mice (6-17x fold for mice at >730, relative to 60-90, days old), due to exhaustion/senescence (21, 25, 46, 47), their levels were not elevated in the BM of Chow-fed mice of either sex by day 500 in our study (**Figures 5A, B**). However, and consistent with the ability of obesity to accelerate ageing, the levels of LSK-HSCs observed in the BM from ageing male and female HCD-fed mice were significantly higher than those of their Chow-fed counterparts by this time point (**Figures 5A, B**; males, ~2.5x fold; females, ~3x fold). Exposure to ES-62 did not significantly modify these elevated levels of HSCs in either male or female HCD-fed mice (**Figures 5A, B**).

Although the d500 Chow-fed mice did not exhibit elevated levels of LSK-HSCs, BM from the Chow-fed animals showed an increased myeloid/lymphoid cell ratio that was evident by d340 in both male and female chow-fed mice (**Figures 5C, D**), findings indicative of the well-established ageing-associated myeloid skewing. This myeloid bias was not modulated by HCD in either male or female animals but interestingly, showed sexual dimorphism in terms of the impact of exposure to ES-62 (**Figures 5C, D**). Thus, whilst ES-62 acted to suppress the myeloid bias in ageing male mice, it perhaps surprisingly strongly promoted it in female HCD-fed animals at d500 (**Figures 5C, D**). The protective effect of ES-62 against this myeloid bias in male mice reflected both a limiting of the HCD-induced decline in B cells (**Figure 5E**) and associated increases of various myeloid lineages (**Figures 6A, E, H**). By contrast, and perhaps consistent with the adipocyte skewing away from the B cell-promoting osteoblastic (or their mesenchymal progenitors) niche (48–50) observed in female mice (**Figures 4H, I**), exposure to ES-62 did not impact on the profound HCD-accelerated decline in B cells (**Figure 5F**) and resulted in enhanced levels of certain myeloid cells in the BM of d500 HCD-fed female animals (**Figures 6B, F**). These differential effects of ES-62 on the ageing-associated myeloid bias in male and female HCD-fed mice were broadly corroborated in terms of absolute numbers of the relevant cell populations (**Supplementary Figure 4**).

Although ES-62-treated HCD-fed mice also exhibited less myeloid/lymphoid skewing than their Chow-fed counterparts, these effects of ES-62 appear generally to be restricted to the accelerated inflammation-induced pathology associated with HCD-feeding. Thus, in separate cohorts of Chow-, HCD- and ES-62-Chow (cES-62)-fed mice (PBS treated, HCD-fed mice were also included as obesity-accelerated ageing controls), we found that treatment with the helminth product had no effect on the BM levels of LSK-HSCs or any myeloid/lymphoid bias pertaining at the d340 time point in the male Chow-fed animals (**Supplementary Figures 5A-D**). By contrast, the d340 female HCD-fed mice exhibited reduced levels of B and T cells, providing corroboration of the sexual dimorphisms in haematopoiesis we have identified during ageing of HCD-fed mice (**Supplementary Figures 5E-L**).

### ES-62 and lymphoid cells

Whilst male Chow- and HCD-fed mice showed a comparable substantial age-associated decline in BM B cells that was significantly mitigated in the ES-62-treated HCD cohort, the reduction observed in female Chow-fed mice was profoundly accelerated in both the PBS- and ES-62-treated HCD cohorts (**Figures 5E, F**). By contrast, levels of BM T cells were relatively stable in all cohorts, although treatment with ES-62 tended to enhance their levels, relative to those exhibited by their PBS-HCD- and Chow-fed male, but not female, counterparts, at d500 (**Figures 5G, H**). Perhaps, given the evidence that RANKL may provide a crucial autocrine factor for B cell development (39), the inverse effects of ES-62 on the proportions of B cells expressing RANKL (**Figures 5I, J**) provide a potential mechanism contributing to its prevention of this myeloid bias in male, but not female HCD-fed mice.

Despite the observed age-associated decline in BM B cells in Chow and HCD-fed male and female mice (**Figures 5E, F**), we found the total levels of splenic and MLN CD19^+^ B cells to increase with age, and this was accelerated and exacerbated by HCD-feeding in male animals, but not modulated by ES-62 in either sex (**Supplementary Figures 6A-D**). Rather, ES-62 increased the levels of “regulatory” IL-10 producing B cells (Bregs) in the spleens (**Supplementary Figure 6E**) and MLNs (11) of HCD-fed male, but not female mice. These “regulatory” actions of ES-62 were again restricted to the HCD-fed animals as its administration to Chow-fed animals was not associated with any significant change in the levels of MLN or splenic Bregs or the levels of CD19^+^CD21^-^CD23^-^CD11c^+^ ageing-associated B cells (ABCs) in the spleens of such animals (**Supplementary Figures 6F-H**). Likewise, ES-62 had little or no effect on naïve and memory CD4^+^ and CD8^+^ T cell populations in the blood, spleen or MLNs of Chow- or HCD-fed mice of either sex (**Supplementary Figures 7-9**).

### ES-62 and myeloid lineages

Neutrophils constitute a major myeloid population in the BM and it has been proposed that the hyperglycemia and hyperlipidemia resulting from HCD/obesity induce enhanced BM production of (primed) neutrophils that contribute to adipose tissue inflammation and development of diabetes and cardiovascular conditions (51). Consistent with previous findings that neutrophil levels are broadly similar in the BM of young and old healthy mice (52), we found that BM neutrophil levels in male Chow-fed mice rise only marginally with age. Whilst this was also true of PBS-HCD-fed male animals, this ageing effect was somewhat countered in the ES-62-treated HCD cohort at d500 (**Figure 6A**). Moreover, in female mice there was no overall change in the levels of neutrophils in Chow- and ES-62-HCD-fed animals over the time course of the experiment, although a significant decrease in their levels was observed in the BM of PBS-HCD-fed female mice at d500 (**Figure 6B**). Likewise, the levels of BM monocytes showed only limited changes during ageing of male and female mice, with HCD-feeding both reducing (d340) and enhancing (d500, albeit not significantly in the case of the female animals) the levels of these cells, regardless of exposure to ES-62 (**Figures 6C, D**). Deeper analysis revealed that the classical, inflammatory Ly6C^hi^ sub-population of monocytes did not exhibit the d500 increase in any of the cohorts of HCD-fed mice (**Figures 6E, F**), perhaps suggesting the HCD-induced decrease (in both total and Ly6C^hi^ monocytes) at d340 reflected the mobilisation of these monocytes associated with obesity-programming of peripheral inflammation.

ES-62 also impacted on the megakaryocyte component of the ageing-associated skewing towards the myeloid lineages that is promoted by the obesity-driven BM adipocyte bias and contributes to the production of platelets with increased inflammatory and thrombotic activity recently implicated in the development of cardiovascular comorbidities (53). Histological analysis revealed increased numbers of megakaryocytes evident even in the bones of ageing Chow-fed mice. Whilst male mice fed a HCD diet displayed significantly more of these cells at d340 and d500, these were reduced by exposure to ES-62 towards the levels seen in Chow-fed animals and a similar modulatory pattern was observed in female mice, particularly at d500 (**Figures 6G–I**). Megakaryocytes have also recently been implicated in promoting HSC quiescence during ageing (49, 50) and thus, together with its enhancement of B and T lymphocytes and decrease in neutrophils, the ES-62-mediated suppression of the rise in megakaryocytes likely contributes to its ability to block the myeloid/lymphoid bias exhibited in the BM of ageing male HCD-fed mice (**Figure 5C**).

Although the ageing myeloid bias is associated with chronic low-grade inflammation in terms of systemic IL-1β and TNFα levels (22), there was no clear pattern regarding effect of sex, HCD or ES-62 on the serum levels of these cytokines across our mouse cohorts. Perhaps of more relevance in the context of obesity, however, increased levels of inflammatory CD11c^+^ macrophages were evident at d340 in the gonadal fat of male PBS-HCD, but not ES-62-HCD-, fed male mice (**Figure 6J**), with the protective effect of ES-62 correlating with a reduction in the mRNA levels of IL-1β and TNFα (**Figures 6K, L**) in this tissue. Experiments involving additional d340 cohorts of PBS-Chow-, ES-62-Chow- and, as an obesity-accelerated ageing control, PBS-HCD-fed mice, showed that treatment with ES-62 had no effect on the levels of these adipose CD11c^+^ macrophages or Bregs in either male Chow-fed mice (**Figures 6M, N**). However, they corroborated the finding that enhanced levels of CD11c^+^ macrophages are found in the gonadal fat pads of male, but not female, HCD-fed mice relative to their Chow-fed counterparts (**Figure 6M**) and in addition, identified HCD-induced depletion of Bregs from this adipose tissue in female, but not male, mice at d340 (**Figure 6N**).

### Small molecule analogues (SMAs) of ES-62 target trabecular bone and the BM niche

Although the findings reported above have potential translational impact, ES-62 itself is not suitable for drug development as it is a large, “foreign” and hence immunogenic glycoprotein. Rather, we have addressed translational potential by designing a library of SMAs of which at least two (11a, 12b) mimic ES-62’s primary mechanism of action in downregulating MyD88 and also recapitulate many of its protective effects in mouse models of chronic inflammatory disease including asthma, arthritis and SLE (10, 32, 54). Crucially, treating mice with 11a plus 12b (combined due to subtle but complementary differences in their immunomodulatory actions), also promotes their healthspan by improving gut and metabolic homeostasis in the HCD model of obesity-accelerated ageing (30). We therefore investigated whether such treatment could recapitulate the impact of ES-62 on the bone microenvironment, focusing on the d160 time-point when much of the (obesity-accelerated) loss of trabecular bone structure was already apparent (**Figures 2**, **3**; **Supplementary Figure 2**). Again, although no significant differences in articular joint pathology (**Figure 7A**) were determined, the profound loss of trabecular bone architecture in all cohorts of female mice and the HCD-acceleration of such pathology in male mice (**Figures 2**, **3**; **Table 1**; **Supplementary Figure 2**) was confirmed. µCT analysis of these d160 cohorts indicated that treatment with the SMA combination clearly mimicked the actions of ES-62 in protecting against trabecular bone loss (**Figures 7B**, **8**), again by preventing the decline in % BV/TV, Tb.N and intersection surface (**Figures 8A, B, J**) and the increase in T.PF and SMI in male, but not female, HCD-fed mice (**Figures 8L, O**).

Such trabecular bone loss and/or its protection by exposure to the SMAs, was again not associated with any substantial effects on the levels of OCPs or their osteoclastic potential (**Supplementary Figures 10A-G**) and consistent with this, there were no significant differences amongst the cohorts in their BM expression of RANK, RANKL or OPG. However, the elevated levels in BM adipocytes generally observed in female, relative to male, mice at d340 and d500 (**Figures 4G–I**) were also evident at the d160 timepoint, although these were not further increased by HCD-feeding (**Figures 7C–E**). Moreover, whilst HCD-feeding increased the levels of BM adipocytes in male mice at this timepoint, this enhancement was countered by their exposure to the SMAs (**Figures 7C, D**). Collectively, these data suggest that trabecular bone loss at this stage may also predominantly reflect dysregulation of BM adipocyte homeostasis and that this regulatory checkpoint exhibits sexual dimorphism in its ES-62/SMAs responsiveness.

Although there were no significant differences between the groups in terms of LSK-HSCs, myeloid/lymphoid skewing or various myeloid lineages in either sex at d160 (**Supplementary Figures 11A, B, E-H**), the HCD-induced decrease in B and T cells in the BM of female mice (**Supplementary Figures 11C, D**) likely reflects adipocyte disruption of the osteoblastic/CXCL12 abundant reticular (CAR) niches that support lymphoid lineages. However, the levels of B and T cells in the blood, spleen or MLNs were not modulated by SMA treatment in either sex (**Supplementary Figure 12**; **Supplementary Figures 13A-F**). Interestingly, given the inflammatory (CD11c^+^ macrophages and IL-1β) nature of adipose tissue in male HCD-fed mice that we identified at d160 (11) and d340 (**Figures 6J–L**), we have also found significant rises in TNFα (at the mRNA level) in the gonadal fat pads of male but not female HCD-mice, which were not reduced by exposure to either ES-62 or SMAs at d160 (**Supplementary Figures 13G, H**). However, whilst the mRNA levels of IL-1β likewise appear to be elevated in these fat pads of male HCD-fed mice at this time point, in this case, ES-62 appears to be able to reduce production of this cytokine in this adipose tissue. A similar pattern tended to be observed in the female animals, although this did not reach statistical significance (**Supplementary Figure 13I**). Nevertheless, collectively these data indicate that the SMAs mimic ES-62’s ability to retrain the BM microenvironment and that their anti-inflammatory actions are associated with their capacity to promote healthspan during obesity-accelerated ageing (11, 30).

## Discussion

Collectively, our analyses of the impact of HCD-feeding on the BM microenvironment and consequent skewing of immune responses across the life-course in male and female C57BL/6J mice underlines the central role that dysregulation of the osteoimmunology axis plays in driving inflamm-ageing and associated metabolic comorbidities, loss of skeletal health, frailty and (obesity-induced acceleration of) the ageing process *per se* (15–20). Moreover, they have highlighted pronounced sexual dimorphisms in these processes with female mice exhibiting more dramatic dysfunction in each of loss of skeletal health, disruption of the BM niche and consequent haematopoiesis towards a more pro-inflammatory phenotype, factors likely contributing to the observed sex differences in the immune system with ageing, particularly with respect to development of autoimmunity and in addition, efficacy of vaccination and cancer immunotherapy (55). Further to this sexual dimorphism, exposure to ES-62 predominately ameliorates such dysregulation in male HCD-fed mice, a differential effect perhaps contributing to our observation that whilst it can promote healthspan in both sexes of obese mice, it is only able to extend the median lifespan of male HCD-fed mice (11).

Such protection in male HCD-fed mice appears to reflect that ES-62 harnesses its immunomodulatory properties to counteract the aberrant training of BM progenitors triggered by the chronic low-grade inflammation associated with obesity, rather than acting on the ageing processes within these cells, *per se*, as it had little effect on the, albeit more limited, dysregulation of haematopoiesis evidenced by Chow-fed mice at 340 days of age. Nevertheless, in reducing the myeloid/adipocyte skewing and loss of BM B cells associated with both “normal” and obesity-accelerated ageing (17, 21–25) in male mice, ES-62 acts to maintain a BM phenotype more consistent with that of young mice. Thus, as it is increasingly evident that all ageing characteristics do not (i) occur synchronously, or indeed linearly, (ii) exhibit sexual dimorphism and (iii) are impacted by acute and chronic exposure to environmental factors such as diet and smoking (56–60), any clear protective effects of ES-62 against dysregulation of the osteoimmunology axis in Chow-fed male mice might not be apparent until a much later age. ES-62 might therefore exhibit protection in much older Chow-fed mice when the “biological” (rather than chronological) ages of key functional responses of such animals correspond to those of younger HCD-fed mice and/or the eventual chronic inflammation resulting from gut dysbiosis and loss of barrier integrity in Chow-fed animals impacts on the ageing process (11–13). Hence, whilst our data indicate a dramatic increase in the levels of HSCs between d340 and d500 in HCD-fed animals, previous studies on C57BL/6 mice showed that Chow-fed mice typically exhibited elevated levels of HSCs displaying loss of functionality between 18-24 months (d547-730), at which point such dysregulation was not further enhanced in mice experiencing a lifelong HCD (61, 62). Interestingly, whilst calorie restriction reduced these elevated HSC levels, it had no effect on their functionality in these old mice (61–63). By contrast, reduced myeloid/lymphoid skewing, improved HSC quiescence and repopulating capacity (partially reversed by insulin-like growth factor-1) has been reported in calorie-restricted “middle age” (9 months old) mice (64). However, perhaps surprisingly therefore, these “middle-aged” calorie-restricted mice also demonstrated suppressed lymphoid differentiation (rescued by provision of IL-6/IL-7), resulting specifically in decreased B cell immunity (64). Collectively, these studies emphasise the lack of correlation/synchronicity amongst various ageing parameters and the disconnect between biological and chronological age (56–60). It is also worth noting, given the profound sexual dimorphisms we have uncovered in the osteoimmunology axis, that these previous studies were either performed on undefined (61, 64) or mixed male and female (63) cohorts, potentially confounding the interpretation of at least some of these functional responses.

Alternatively, and consistent with our wealth of data suggesting that ES-62 acts to resolve chronic inflammation rather than suppressing steady-state or “emergency” responses, it is possible that ES-62 only targets “hyper-responsive” cells, the cross-talk between signals generated by ES-62 and the pathogenic microenvironment generating a unique, modulated phenotype. Supporting this idea, whilst ES-62 similarly has little or no effect on the functionality of fibroblasts in the synovial joints of healthy mice, it induces a stable epigenetically rewired, inflammation-resolving/tissue repair phenotype of synovial fibroblast in mice undergoing collagen-induced arthritis (CIA; a model of rheumatoid arthritis), that is distinct from that found in naïve animals (65). Interestingly, IL-1β signalling, a key factor in driving the pathogenic transformation of synovial fibroblasts in CIA and rheumatoid arthritis that is targeted by ES-62 (65), is crucial to the pro-inflammatory training of both BM and migratory “surveillance” haemopoietic stem and progenitor cells (HSCPs) and their resultant accumulation and differentiation into adipose tissue-resident myeloid cells that perpetuates the inflammatory osteoimmunology cycle in obesity (24, 66–68). Pertinent to this, we have now shown the levels of IL-1β, TNFα and CD11c^+^ inflammatory macrophages to be reduced in the gonadal adipose tissue of male, but not female, HCD-mice by ES-62 at d340. Associated with this, we have previously reported that the helminth product reduces the levels of pro-inflammatory CD11c^+^ macrophages whilst increasing those of anti-inflammatory CD11c^-^CD301^+^ macrophages found in these fat depots at d160 and in addition, counteracts the elevated levels of IL-1β in the liver in male, but not female HCD-fed animals at d500 (11).

ES-62’s key actions in combating dysregulation of the osteoimmunology axis in male HCD-fed mice focus on slowing the ageing-related myeloid/lymphoid bias and associated loss of B lineage cells, as well as protecting the BM niche by suppressing the mesenchymal skewing towards adipocyte accumulation. These actions are likely interconnected as adipocyte skewing results in the depletion of the osteoblasts (or progenitors) proposed to be important, *via* their generation of IL-7 and CXCL12 (SDF-1), for B cell differentiation (39), as well as disrupting the BM niche and inducing pathogenic bone remodelling (25, 69–71). Given the increasing evidence that LPS-stimulated TLR4/MyD88 signalling is critical in driving both emergency pro-inflammatory myelopoiesis to fight infection and the myeloid/lymphoid skewing associated with (obesity-accelerated) ageing (66), our working model is that ES-62 harnesses its ability to subvert TLR4 signalling and downregulate MyD88 in order to counteract such dysregulation of HSCs, both in the BM and the periphery. Moreover, the central role of sensing of LPS (66), elevated in serum as a consequence of microbiota dysbiosis and loss of gut barrier integrity in each of infection and obesity, conditions associated with chronic inflammation and ageing *per se*, underlines the key contributions of the TLR4/MyD88 signalling cassette and the gut-osteoimmunology axis in the (dys)regulation of health and well-being over the life-course (11–14, 17–19, 25, 69–71).

Strikingly, impairment of HSC function has now been reported to occur prior to the onset of obesity-induced accumulation of adipose tissue CD11c^+^ macrophages and the inflammation associated with the IL-1β- and TNFα-driven myeloid/lymphoid skewing of BM progenitors (24). This led to the discovery that LPS impacts directly on the functionality of (TLR4 expressing) BM HSCs (66), with deeper analysis revealing MyD88 signalling to be responsible for expansion of granulocyte/macrophage precursors and contributing to the accumulation of adipose tissue macrophages (72). LPS-TLR4/MyD88 signalling likewise similarly impacts on (migratory) Haematopoietic Stem and Progenitor Cells (HSPCs) (67), mobilised to the periphery in response to obesity where they also differentiate and accumulate as adipose tissue macrophages (68) to perpetuate inflammation and, *via* IL-1β release, further biasing of differentiation of BM cells (24, 66). HCD-depletion of the Common Lymphoid Progenitor (CLP) and B cell-differentiation-promoting CXCL12 abundant reticular (CAR) cells in the BM (25) additionally reinforces the myeloid/lymphoid bias (66) as does the HCD enhancement of Nestin^+^ MSCs, which drive the adipocyte bias of the BM niche in obesity (25). Further to the osteoimmunology axis-targeting actions of ES-62 in the BM, MSCs also express TLRs and whilst LPS promotes differentiation of the proinflammatory (IL-6/IL-8-producing) MSC1 subset, by contrast TLR3 signalling promotes differentiation of an immunosuppressive MSC2 subset (73, 74). Intriguingly therefore, as ES-62 can desensitize TLR4/MyD88 but not TLR3 signalling (10), by shifting the TLR4/TLR3 and hence MSC1/MSC2 functional balance it could effectively reset homeostasis of the osteoimmunology axis to promote healthspan. Certainly, TLR3 agonists are being explored for their potential to repair the BM niche and direct MSC2 to repair cardiac damage and cardiovascular disease in obesity (75–77).

Interestingly, MyD88 has also proven important in the regulation of functional responses of B cells in the periphery as, whilst MyD88-deficient B cells were impaired in upregulation of CD86 and proliferation, they exhibited increased synthesis of DNP-specific IgG1 antibodies (78), an isotype consistent with the modified (regulatory) TH2 immune response to ES-62 (79). Likewise, B cell depletion of MyD88 results in abrogation of pathogenic autoantibody responses (80–82) and is associated with the ES-62-induction of Bregs in mouse models of systemic lupus erythematosus, asthma and RA (14, 83, 84). Notably, treatment of HCD-fed male mice with ES-62 results in increased levels of splenic Bregs, reflecting our previous report of a similar induction of Bregs in the MLNs of these animals (11). Interestingly therefore, adoptive transfer of a splenic CD9^+^ Breg population derived from mice infected with the parasitic trematode *Schistosoma japonicum* has recently been shown to promote metabolic health and suppress inflammation in young female C57BL/6 mice acutely fed (from 4 weeks of age) a high fat diet for up to 9 weeks (85). Moreover, whilst ES-62 also results in the upregulation of ABCs (high PC-reactivity) and anti-PC antibodies, this is likely to be beneficial in countering the declining immunity to (PC-containing) pneumococcal infection in ageing (HCD-fed) males (11, 86). Such natural antibodies have also been implicated in the induction of Bregs (87, 88), which by interacting with (dietary, endogeneous and microbiota-derived) lipid-sensing NKT cells, potentially provide an additional mechanism to counter the chronic pathogenic inflammation occurring in autoimmunity (89, 90) and obesity-accelerated ageing (11).

Nevertheless, despite even our young (d56) female Chow-fed mice exhibiting profoundly disrupted trabecular bone structure that presumably predisposes them to potentially irreversible dysregulation of the BM niche and consequent immune responses, our TLR4/MyD88-targeting mechanistic model leaves the key question, of why the osteoimmunology axis of female HCD-fed mice is relatively unresponsive to ES-62, unanswered. Indeed, given that our original hypothesis was that ES-62 would combat the chronic inflammation that causes retraining of BM progenitors, it might have been predicted that it would be more effective in female mice as they typically make stronger inflammatory responses than male animals (55). However, we found some surprising data with, for example, female HCD-fed mice exhibiting the lowest levels of neutrophils in the BM at d500: whilst this may simply reflect their mobilisation to the periphery under the conditions of chronic inflammation associated with obesity (91), it is also possible that these reduced levels could be due to the loss of CD62L^lo^CD11b^hi^ neutrophils that are a subpopulation of myeloid suppressor cells that normally home to the BM (92). Likewise, the highest numbers of adipocytes accumulated in the BM were observed in Chow- and ES-62-HCD-fed female mice at d500. However, increasing evidence that BM adipose tissue (MAT) functions as an endocrine organ (and hence may be differentially responsive to sex hormones) that can promote metabolic adaptation and bone homeostasis, whilst generally exerting detrimental effects on haematopoiesis and osteogenesis (93, 94), makes these findings more complicated to interpret without deeper characterisation of their functional phenotype. For example, BM accumulation of adipocytes can be a stress response acting to protect cells from lipotoxicity and providing a mobilisable reservoir during energy deficit (95), with brown MAT driving the energy balance, adaptive thermogenesis and releasing IGF-1 and leptin, factors that promote osteogenesis and bone mass (95). By contrast, sensing of obesity-induced oxidative stress could disrupt the brown/white MAT balance, leading to further release of cytokines and free fatty acids perpetuating inflammation (95–97). Intriguingly therefore, a novel bone-specific, ageing-associated adipogenesis pathway has recently been identified that, under conditions of metabolic stress, results in the accumulation of lipid-storing adipocytes within the haematopoietic niche, particularly in female mice (98).

Turning once again to osteogenesis, in terms of the proposed roles of leptin and adiponectin in promoting this [by increasing osteoblastic and suppressing osteoclastic activities (99–101)] and modulating haematopoiesis and inflammatory responses in mouse models, we had previously measured the effects of exposure to ES-62 on serum levels of these adipokines in our cohorts of ageing male and female mice (11). As obesity induces bone loss, it was not surprising that we found HCD-feeding to suppress serum adiponectin levels in both sexes. However, and perhaps rather unexpectedly from an osteogenic viewpoint, obesity was found to be associated with enhanced leptin levels. Nevertheless, this may simply reflect that chronic exposure to HCD induced a state of leptin resistance in these animals (11). Moreover, whilst ES-62 did not significantly reduce the HCD-induced gain in body mass and had no effect on HCD-associated adiponectin levels, it suppressed serum leptin levels in both sexes of HCD-fed mice at d500 (11). These data perhaps suggest that rather than targeting adipokine levels to promote osteogenesis, ES-62 might be acting to counter the inflammatory metabolic actions of leptin at this time point. Alternatively, as leptin can act directly on MSCs to promote BM adipogenesis (102), the reduction of serum leptin by ES-62 could contribute to the significant reduction of adipocyte skewing and disruption of the bone marrow osteoblastic niche observed in male HCD-fed mice at d500. However, once again, sexual dimorphism is evident, as this adipocyte reduction is not found in female animals.

Thus, to further dissect the mechanisms underpinning the sexual dimorphism in ES-62 -protection of bone health and the haematopoiesis-supporting BM niche, we suggest that extensive bone histomorphometry (103) and measurement of more definitive serum biomarkers (104) of bone resorption (e.g., CTX-1) and formation (e.g., P1NP) could be employed. These techniques could help determine how the structural changes in the bones of our ageing mouse cohorts determined by µCT relate to dysregulation of the OC/OB balance controlling bone resorption and formation. Complementing these approaches, we aim to gain a fuller understanding of the divergent functional rewiring of BM populations underlying the sexual dimorphisms in dysregulation of haematopoiesis induced by HCD-accelerated ageing and their differential ES-62 responsiveness. To achieve this, we are currently embarked on a program of RNAseq (single cell and bulk) and adoptive transfer/reconstitution *in vivo* studies which, in concert with bioinformatic analyses, aims to identify the key functional and cellular BM phenotypes involved. Certainly, knowledge of these ageing BM phenotypes is imperative for exploiting the potential for ES-62 (and other interventions) to differentially target inflammatory and stromal cells (and progenitors) in the BM and periphery, to tailor inflammatory responses appropriate to the sexual dimorphisms evolved by the immune system to promote the health and lifespan of male and female individuals.

Finally, in terms of translating these actions of ES-62 to the clinic, we are encouraged by the ability of the SMAs to mimic not only its key gut and metabolic effects (30) but also certain of its impacts on the osteoimmunology axis, most notably in strong protection of the trabecular bone structure and BM niche. Such protection augurs well for their potential as starting points in the identification of novel ES-62-based anti-ageing interventions, particularly those targeting osteolytic diseases and chronic inflammatory disorders and comorbidities. Moreover, these studies once again underline the importance of targeted drug development in terms of addressing the sexual dimorphisms associated with ageing and inflammation-based comorbidities.

## Data availability statement

The original contributions presented in the study are included in the article/**Supplementary Material**. Further inquiries can be directed to the corresponding authors.

## Ethics statement

The animal study was reviewed and approved by University of Glasgow Animal Welfare and Ethical Review Board.

## Author contributions

JD, FL, JC, ROD, GB, JD-M, and DW performed the experiments and/or contributed to the analysis of the data for the study that MH, WH, and CS conceived. JD and FL prepared ES-62 and CJS produced the ES-62-based SMAs. MH, WH, and CS supervised the study and experimental design and contributed to data analysis. MH and WH prepared the manuscript and all authors were involved in its review and revision and have approved the final version.

## Funding

This work was funded by awards to MH, WH and CS from the BBSRC (BB/M029662/1, BB/M029727/1, BB/V001027/1 and BB/V000993/1).

## Acknowledgments

The authors would like to thank Kevin Mackenzie at the Microscopy and Histology Core Facility at the Institute of Medical Sciences, University of Aberdeen for his help in the processing and analysis of the femur microCT samples.

## Conflict of interest

The authors declare that the research was conducted in the absence of any commercial or financial relationships that could be construed as a potential conflict of interest

## Publisher’s note

All claims expressed in this article are solely those of the authors and do not necessarily represent those of their affiliated organizations, or those of the publisher, the editors and the reviewers. Any product that may be evaluated in this article, or claim that may be made by its manufacturer, is not guaranteed or endorsed by the publisher.
