# Supplementary material for: The parasitic worm product ES-62 protects the osteoimmunology axis in a mouse model of obesity-accelerated ageing
Source: Front Immunol. 2022 Aug 29;13:953053. doi: 10.3389/fimmu.2022.953053 (PMC9465317; doi:10.3389/fimmu.2022.953053)

Supplementary Figures

Supplementary Figure 1. Exemplar Gating Strategies. (A-C) Following exclusion of dead cells/cell debris and gating of BM cells (by forward scatter versus side scatter, **A-Ci**), cell doublets were excluded **(A-Cii)** prior to subsequent gating of **(A)** Lin^- populations using a dump channel **(Aiii)** to identify LSK HSCs ($Lin^-Sca\text{-}1^+c\text{-}Kit^+[CD117^+]$, **Aiv**); **(B)** exclusion of lymphocytes and erythroid cell populations using a dump channel comprising a cocktail of antibodies specific for CD3, B220 and Ter119 **(Biii)** and then distinguishing monocytes ($Ly6C^{high}Ly6G^-$) from neutrophils **(Biv)** to identify OCPs ($CD11b^{low}Ly6C^{high}Ly6G^-$) including the $CD115^+$ subpopulation **(Bv)**, that reside within the global monocyte population; **(C)** $CD45^+$ and $CD45^-$ populations **(Ciii)**, with the former, in conjunction with SSC, allowing identification **(Civ)** of B ($B220^+$) and T ($CD3^+$) cells and their respective $RANKL^+$ subpopulations **(Cv & vi)**. **(D)** Splenocytes were gated for lymphocyte morphology (FSC-A vs. SSC-A, **Di**) and then live cells determined by their uptake of the fixable live/dead cell stain live/dead status **(Dii)** and doublets excluded (FSC-H vs. FSC-A, **Diii**) before gating prior to assessing expression of **(Div)** $CD4^+$ T cells; (**Dv**) $CD103^+CD4^+$ T cells and **(Dvi)** $CD8^+$ T cells. **(E)** Blood cells were gated for lymphocyte morphology (FSC-A vs. SSC-A, **Ei**) and then live cells determined by their uptake of the fixable live/dead cell stain live/dead status **(Eii)** before gating prior to assessing expression of $CD3^+$ T cells **(Eiii)** and then $CD4^+$ and $CD8^+$ T cells **(Eiv)** prior to analysis of $CD45RB^+$ and $CD44^+$ on $CD8^+$ T cells (**Ev**) or $CD4^+$ T cells **(Evi)**. All analysis was by reference to relevant isotype or GMO controls.

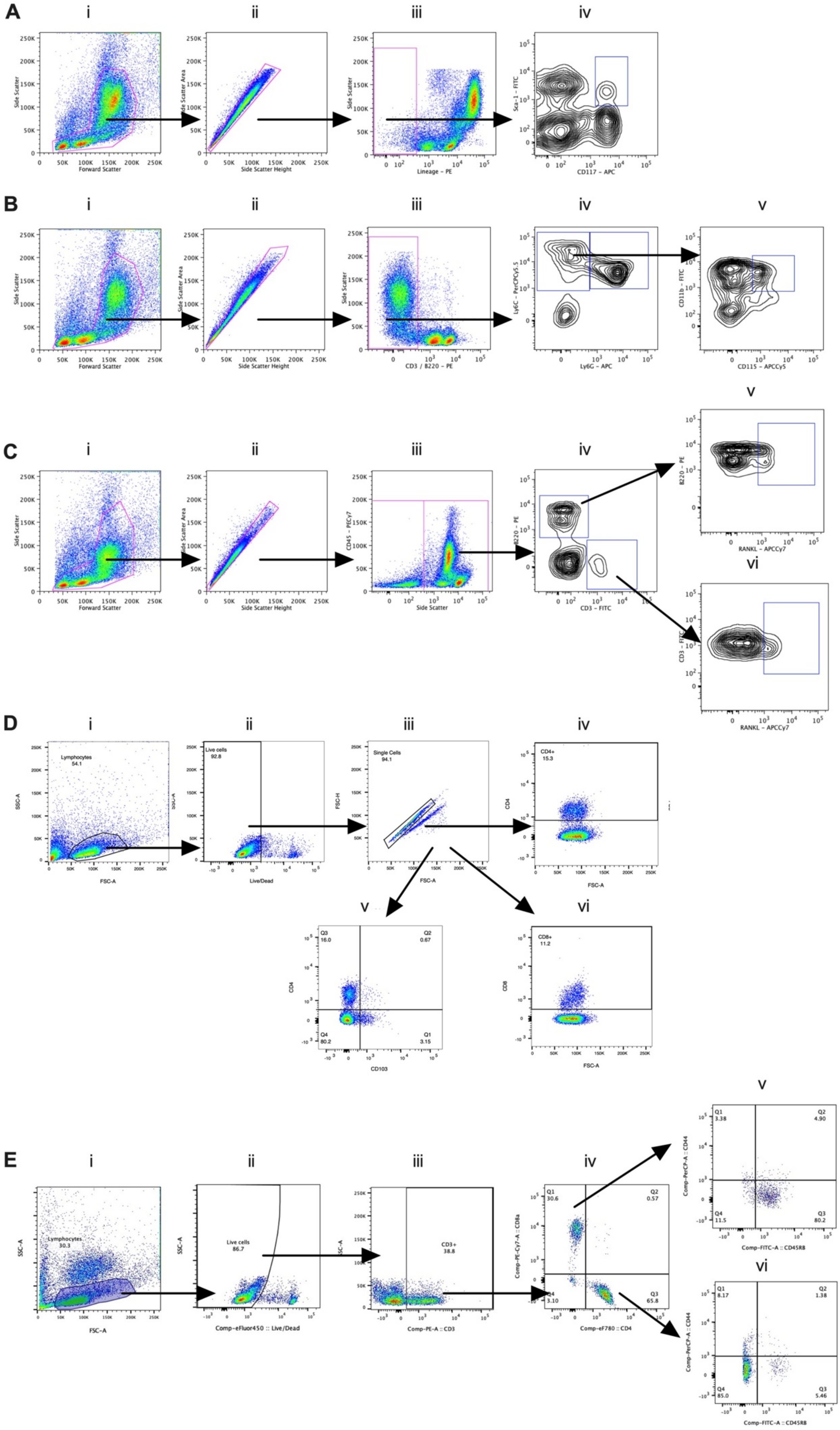
A
B
C
D
E
i
ii
iii
iv
v
vi
Side Scatter
Forward Scatter
Side Scatter Area
Side Scatter Height
Lineage - PE
Sca-1 - FITC
CD117 - APC
CD3 / B220 - PE
Ly6C - PerCPCy5.5
Ly6G - APC
CD11b - FITC
CD115 - APCCy5
CD45 - PECy7
B220 - PE
CD3 - FITC
RANKL - APCCy7
Lymphocytes
54.1
SSC-A
FSC-A
Live cells
92.8
Live/Dead
Single Cells
94.1
FSC-H
CD4+
15.3
CD4
CD103
Q3
16.0
Q2
0.67
Q4
80.2
Q1
3.15
CD8+
11.2
CD8
Lymphocytes
30.3
Live cells
86.7
Comp-eFluor450 :: Live/Dead
CD3+
38.8
Comp-PE-A :: CD3
Q1
30.6
Q2
0.57
Q3
65.8
3.10
Comp-PE-Cy7-A :: CD8a
Comp-eF780 :: CD4
Q1
3.38
Q2
4.90
Q4
11.5
Q3
80.2
Comp-PerCP-A :: CD44
Comp-FITC-A :: CD45RB
Q1
8.17
Q2
1.38
Q4
85.0
Q3
5.46

Supplementary Figure 2. HCD-accelerates changes in parameters of trabecular bone architecture in male mice. The effects of HCD and ageing on homeostasis of the indicated parameters **(A-O)** of femoral bone structure were determined by µCT analysis. The data shown are the mean ± SEM values of femurs from n individual mice where for male mice, at d56: n=6; d160: Chow, n=5, HCD, n=6; d340: Chow, n=6, HCD, n=6 and at d500, Chow, n=5, HCD, n=6 and for female mice, at d56: n=6; d160: Chow, n=6, HCD, n=6; d340: Chow, n=6, HCD, n=6 and at d500, Chow, n=5, HCD, n=6. Significant differences are indicated by black***=p<0.001 for male Chow v male HCD **(A, B, D, E, I, L, N, O)**; black**=p<0.01 for male Chow v male HCD **(E, J, L)**; black*=p<0.05 for male Chow v male HCD **(C, F, J)**; blue*** = p<0.001, male HCD v female Chow and/or female HCD **(A, B, D, E, G-J, L, N, O)**; blue**=p<0.01 for male HCD v female Chow and/or female HCD **(A-J, L-O)**, blue*= p<0.05, male HCD v female Chow and/or female HCD **(A, C, J, L, M, O)**; red***=p<0.001 for female HCD v female Chow **(H)**; red**=p<0.01 for female HCD v female Chow **(F-H, M, N)** and red*=p<0.05 for female HCD v female Chow **(C, G, J, L)** groups. In addition but not shown, across the time-course, p<0.0001 for male Chow v female Chow **(A-E, G-O)** and p<0.001 for male Chow v female Chow **(F)**; p<0.0001 for male Chow v female HCD **(A-F, I-L, N, O)** and p<0.001 for male Chow v female HCD **(M)**; Some of the male and female data sets (at d160 and d340) provide the controls for the ES-62 trabecular bone protection analysis shown in Fig.3.

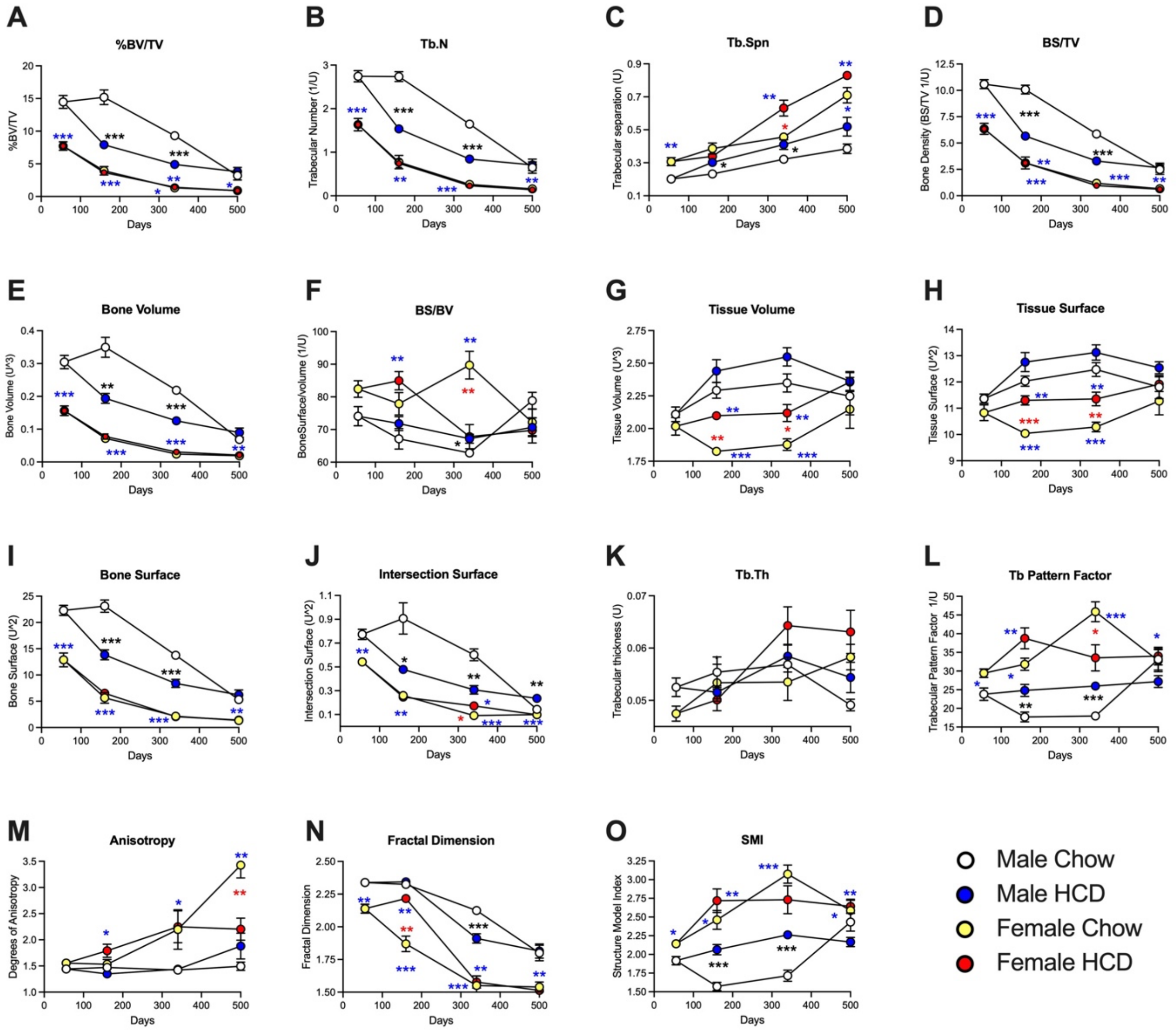

Supplementary Figure 3. Osteoclastogenesis and RANK/RANKL expression in the bone marrow. (A & B) The levels of $CD3^{-}B220^{-}Ter119^{-}Ly6G^{-}Ly6C^{high}$ $CD11b^{low}$ OCPs were determined at d340 as the % live BM cells in **(A)** male and **(B)** female mice fed Chow or HCD, with the Chow cohort treated with either PBS (Chow) or ES-62 (cES-62). The data shown are the values of individual mice (symbols) with the mean value for the group represented by the bar. Significant differences **(B)** are indicated by red****=p<0.0001 for female HCD v female Chow and/or female cES-62. **(C & D)** OC differentiation (at day 5) was assessed by TRAP staining and the number of multinucleated OCs with >3 nuclei determined by Image J analysis with the mean group OC number ± SEM determined at the indicated time points from the mean values of triplicate cultures of n individual male **(C)** and female **(D)** Chow- and HCD-fed mice, the latter cohort treated with either PBS or ES-62. For male mice, at d56: n=6; d340: Chow, n=6, PBS, n=3 and ES-62, n=4; and at d500, Chow, n=5, PBS, n=5 and ES-62, n=5 and for female mice, at d56: n=6; d340: Chow, n=6 , PBS, n=3 and ES-62, n=4; and at d500, Chow, n=4, PBS, n=5 and ES-62, n=5. **(E-H)** The cell surface expression of RANKL on $CD45^{-}$ cells (**E**, male & **F**, female; MFI) and $CD45^{+}$ cells (**G**, male & **H**, female; MFI) were determined by flow cytometry at the indicated timepoints in male **(E, G)** and female **(F, H)** mice fed Chow or HCD, the latter cohort treated with either PBS or ES-62. The data are presented as the mean ± SEM values for n individual mice in each group, where for male mice, at d56: n=6; d340: Chow, n=6, PBS, n=7 and ES-62, n=8; and at d500, Chow, n=5 , PBS, n=5 and ES-62, n=5 and for female mice, at d56: n=6; d340: Chow, n=6 , PBS, n=8 and ES-62, n=8; and at d500, Chow, n= 3, PBS, n=5 and ES-62, n=6. **(I, J)** The mRNA levels of RANK expression in BM cells from male **(I)** and Female **(J)** mice where the data are presented as the mean ± SEM values for n individual mice in each group, where for male mice, at d56: n=6; d340: Chow, n=6 , PBS, n=7 and ES-62, n=8; and at d500, Chow, n=5 , PBS, n=5 and ES-62, n=5 and for female mice, at d56: n=6; d340: Chow, n=6, PBS, n=7 and ES-62, n=8 ; and at d500, Chow, n= 3, PBS, n=3 and ES-62, n=4. Significant differences are indicated by black*=p < 0.05 for Chow v PBS and/or ES-62 **(J)**; black**=p<0.01 for Chow v PBS and/or ES-62 **(E, J)**, black***=p<0.001 for Chow v PBS and/or ES-62 **(E, G)** and red*=p < 0.05 for female PBS v female ES-62 **(H, J)** groups.

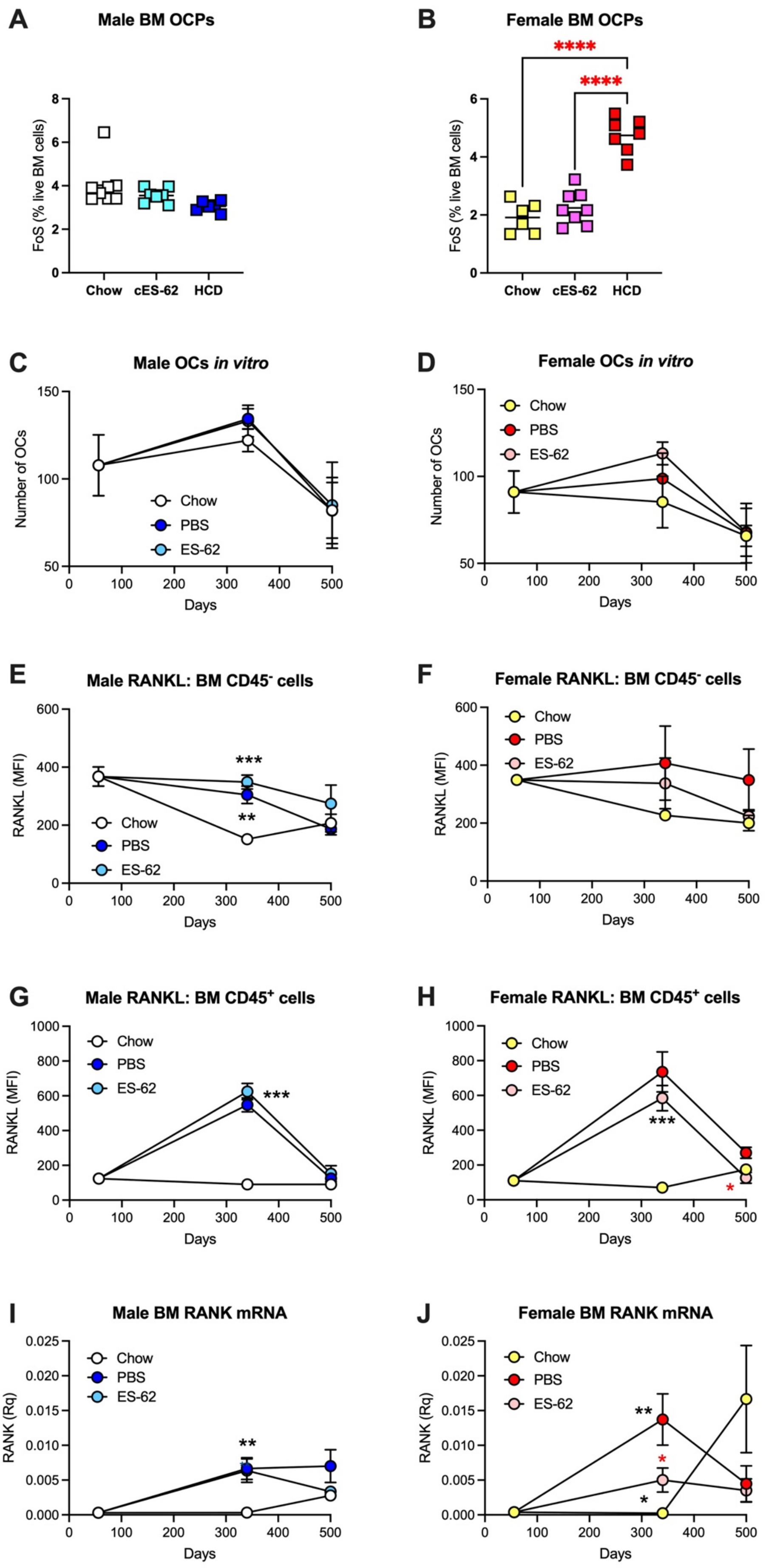
A
Male BM OCPs
FoS (% live BM cells)
Chow
cES-62
HCD
B
Female BM OCPs
****
****
FoS (% live BM cells)
Chow
cES-62
HCD
C
Male OCs in vitro
Number of OCs
Days
Chow
PBS
ES-62
D
Female OCs in vitro
Number of OCs
Days
Chow
PBS
ES-62
E
Male RANKL: BM CD45- cells
RANKL (MFI)
Days
***
**
Chow
PBS
ES-62
F
Female RANKL: BM CD45- cells
RANKL (MFI)
Days
Chow
PBS
ES-62
G
Male RANKL: BM CD45+ cells
RANKL (MFI)
Days
***
Chow
PBS
ES-62
H
Female RANKL: BM CD45+ cells
RANKL (MFI)
Days
***
*
Chow
PBS
ES-62
I
Male BM RANK mRNA
RANK (Rq)
Days
**
Chow
PBS
ES-62
J
Female BM RANK mRNA
RANK (Rq)
Days
**
*
*
Chow
PBS
ES-62

Supplementary Figure 4. Impact of HCD on numbers of BM cell lineages in ageing mice. The numbers of total BM cells **(A & B),** Lin$^-$Sca-1$^+$c-Kit$^+$ (LSK) HSC (**C & D**), OCPs **(E & F)**, CD19$^+$ B cells **(G & H)** and CD3$^+$ T cells **(I & J)** in BM at the indicated time-points from male **(A, C, E, G & I)** and female **(B, D, F, H & J)** mice fed Chow or HCD, the latter cohort treated with either PBS or ES-62. The data are presented as the mean ± SEM values for n individual mice in each group, where for male mice, at d56: n=6; d340: Chow, n=6, PBS, n=7 and ES-62, n=8; and at d500, Chow, n=5, PBS, n=5 and ES-62, n=5 and for female mice, at d56: n=6; d340: Chow, n=6, PBS, n=8 and ES-62, n=8; and at d500, Chow, n= 3, PBS, n=5 and ES-62, n=6. Significant differences are indicated by *=p < 0.05 for Chow v ES-62 and/or PBS **(C, E, H)**; **=p<0.01 for Chow v PBS and ES-62 **(B)** and ***=p<0.001 for Chow v PBS and ES-62 **(C, J)** groups.

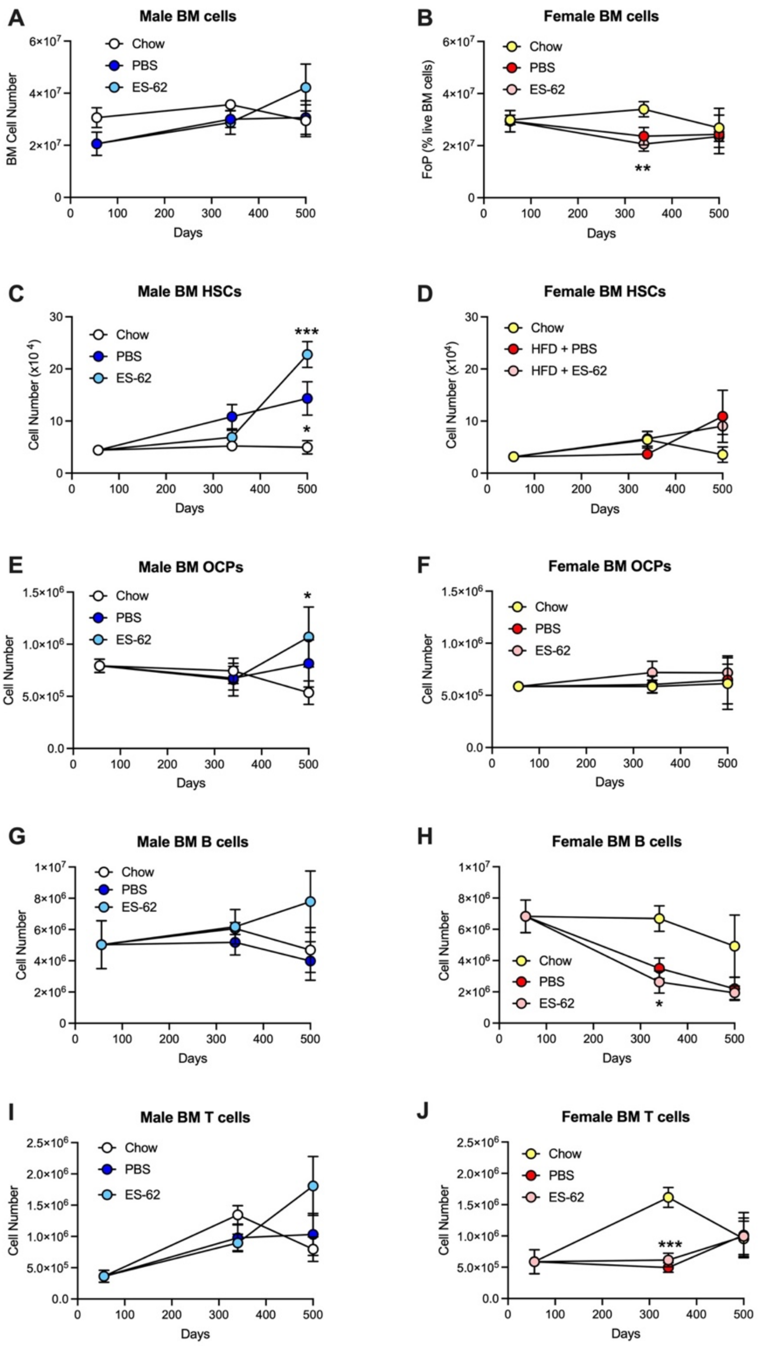

Supplementary Figure 5. ES-62 does not impact on the proportions of BM cell lineages in Chow-fed mice at d340. The levels of Lin$^-$Sca-1$^+$c-Kit$^+$ (LSK) HSC (**A**, male & **B**, female; % Lin$^-$ cells), the ratios of myeloid/lymphoid lineages (**C**, male & **D**, female) and the levels of CD19$^+$ B cells **(E & F)**, CD3$^+$ T cells **(G & H)**, Ly6C^{high} monocytes **(I & J)** and Ly6C$^+$Ly6G$^+$ neutrophils **(K & L)** were determined at d340 as the % live BM cells in male **(A, C, E ,G, I, & K)** and female **(B, D, F, H, J & L)** mice fed Chow or HCD, with the Chow cohort treated with either PBS (Chow) or ES-62 (cES-62). The data shown are the values of individual mice (symbols) with the mean value for the group represented by the bar. Significant differences are indicated by red**=p<0.01 for female HCD v female cES-62 **(B, H)**; red*=p < 0.05 for female HCD v female Chow **(B)**; black*=p<0.05 for Chow v HCD **(F, G)** or cES-62 **(H)**; blue****=p<0.0001 for male HCD v male Chow and/or cES-62 **(I)**; blue***=p<0.001 for male HCD v male Chow and/or cES-62 **(K)** and blue**=p<0.01 for male HCD v cES-62 **(K)** groups.

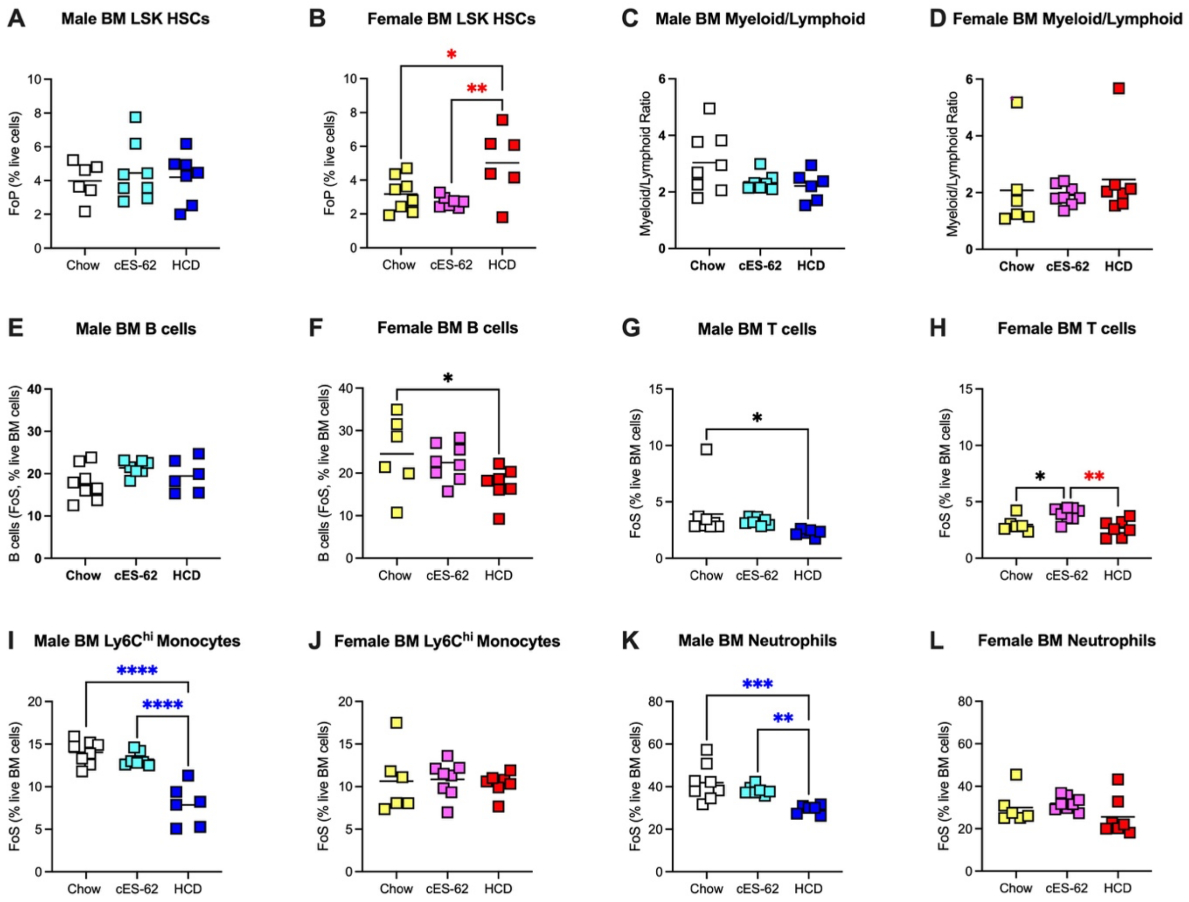

Supplementary Figure 6. Changes in the levels of splenic and MLN B cell populations during obesity-accelerated ageing. The proportions of splenic **(A & B)** and MLN **(C & D)** $CD19^+$ B cells were determined at the indicated times as the % live cells in male **(A & C)** and female **(B & D)** mice fed Chow or HCD, the latter cohort treated with either PBS or ES-62. The data are presented as the mean ± SEM values for n individual mice in each group, where for male mice, d56: n=6; d340, Chow, n=6, PBS: n=10 (7, MLN), ES-62: n=12 (8, MLN); d500: Chow, n=5, PBS, n=6, ES-62, n=6 (6, MLN) and for female mice, d56: n=5 (6, MLN), d340: Chow, n=6, PBS, n= 10, ES-62, n=12 (10, MLN); d500: Chow, n=5 (4, MLN), PBS, n=6 (5, MLN), ES-62, n=6. **(E)** The levels of IL-10^+CD19^+ B cells were determined at d340 as the % live cells in the spleens of male and female mice fed Chow or HCD, the latter cohort treated with either PBS or ES-62. The levels of **(F)** MLN and **(G)** splenic IL-10^+CD19^+ B cells and **(H)** splenic $CD19^+CD21^-CD23^-CD11c^+$ ABCs were determined at d340 as the % live cells in spleens/MLNs of male and female mice fed Chow or HCD, with the Chow cohort treated with either PBS (Chow) or ES-62 (cES-62). The data shown are the values of individual mice (symbols) with the mean value for the group represented by the bar. Significant differences are indicated by ***=p<0.001 for Chow v PBS and ES-62 (**A**); **=p<0.01 for Chow v PBS and ES-62 (**C**) and $^*p < 0.05$ for Chow v PBS and/or ES-62 (**C, E**) groups.

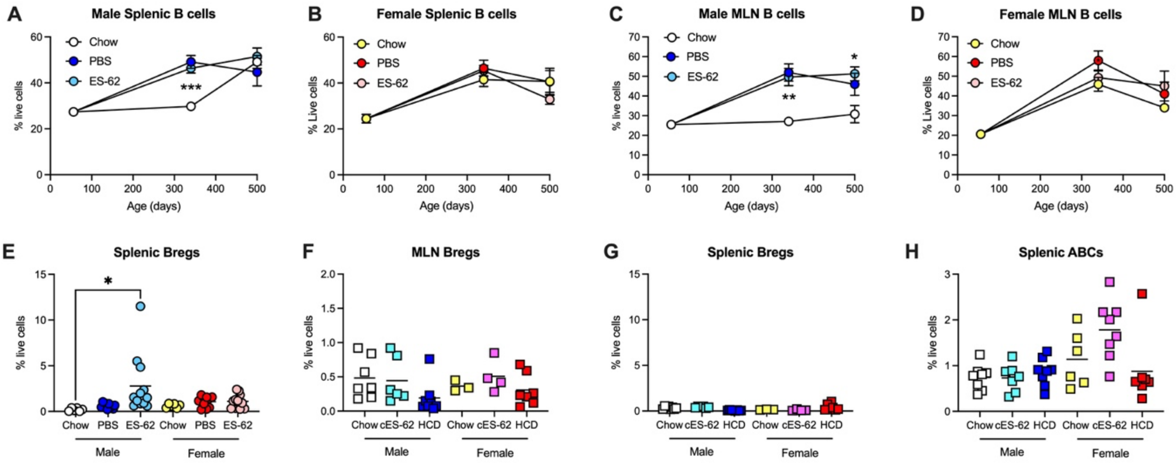

Supplementary Figure 7. Changes in the levels of naïve and memory CD4$^+$ and CD8$^+$ T cells circulating in the blood during obesity-accelerated ageing. The proportions of total CD4$^+$ **(A & B)** and CD8$^+$ **(C & D)** T cells and the levels of naïve (CD45RB$^+$CD44$^-$; **E-H**) and memory (CD45RB$^-$CD44$^+$; **I-L**) CD4$^+$ **(E, F, I & J)** and CD8$^+$ **(G, H, K & L)** T cells were determined at the indicated time-points as the % live cells in male **(A, C, E, G, I & K)** and female **(B, D, F, H, J & L)** mice fed Chow or HCD, the latter cohort treated with either PBS or ES-62. The data are presented as the mean ± SEM values for n individual mice in each group, where for male mice, d56: n=6; d340, Chow, n=6, PBS: n=7, ES-62: n=8; d500: Chow, n=5, PBS, n=6, ES-62, n=6 and for female mice, d56: n=6, d340: Chow, n=6, PBS, n= 7, ES-62, n=8; d500: Chow, n=3, PBS, n=6, ES-62, n=6. Significant differences are indicated by black*=p < 0.05 for Chow v ES-62 and/or PBS **(A, B, J, K, L)**; black**=p<0.01 for Chow v ES-62 and/or PBS **(E, I, J)** and black***=p<0.001 for Chow v ES-62 and PBS **(H)** and blue*=p<0.05 for male PBS vs ES-62 **(I)** groups.

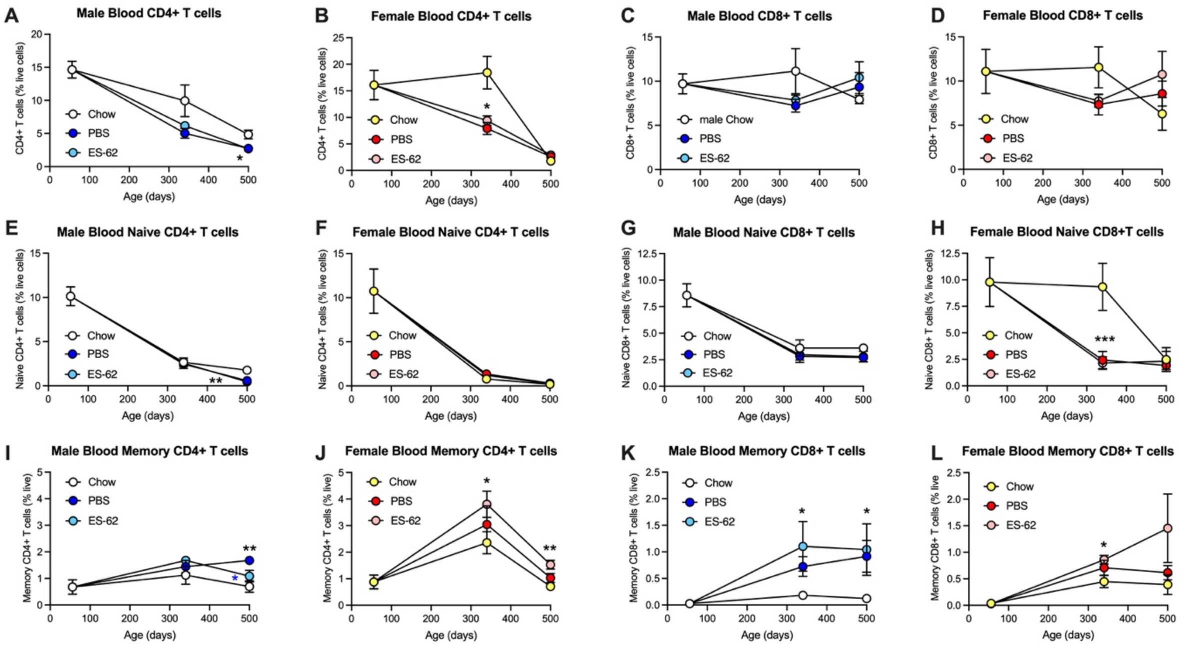

Supplementary Figure 8. Effect of ES-62 on the levels of naïve and memory T cells circulating in the blood of Chow-fed mice at d340. The proportions of total CD4$^+$ **(A & B)** and CD8$^+$ **(C & D)** T cells and the levels of naïve (CD45RB$^+$CD44$^-$; **E-H**) and memory (CD45RB$^-$CD44$^+$; **I-L**) CD4$^+$ **(E, F, I & J)** and CD8$^+$ **(G, H, K & L)** T cells were determined at d340 as the % live cells in the blood of male **(A, C, E, G, I & K)** and female **(B, D, F, H, J & L)** mice fed Chow or HCD, the Chow cohort treated with either PBS (Chow) or ES-62 (cES-62). The data shown are the values of individual mice (symbols) with the mean value for the group represented by the bar. Significant differences are indicated by black*=p < 0.05 for Chow v HCD **(A, K)** or cES-62 **(B, J)**; blue**= p<0.01 for male HCD v male cES-62 **(A)**; blue*=male HCD v male cES-62 **(E, G, K)**; red*=p<0.05 for female HCD v female cES-62 **(B, D)** and red**=p<0.01 for female HCD v female cES-62 **(J)** groups.

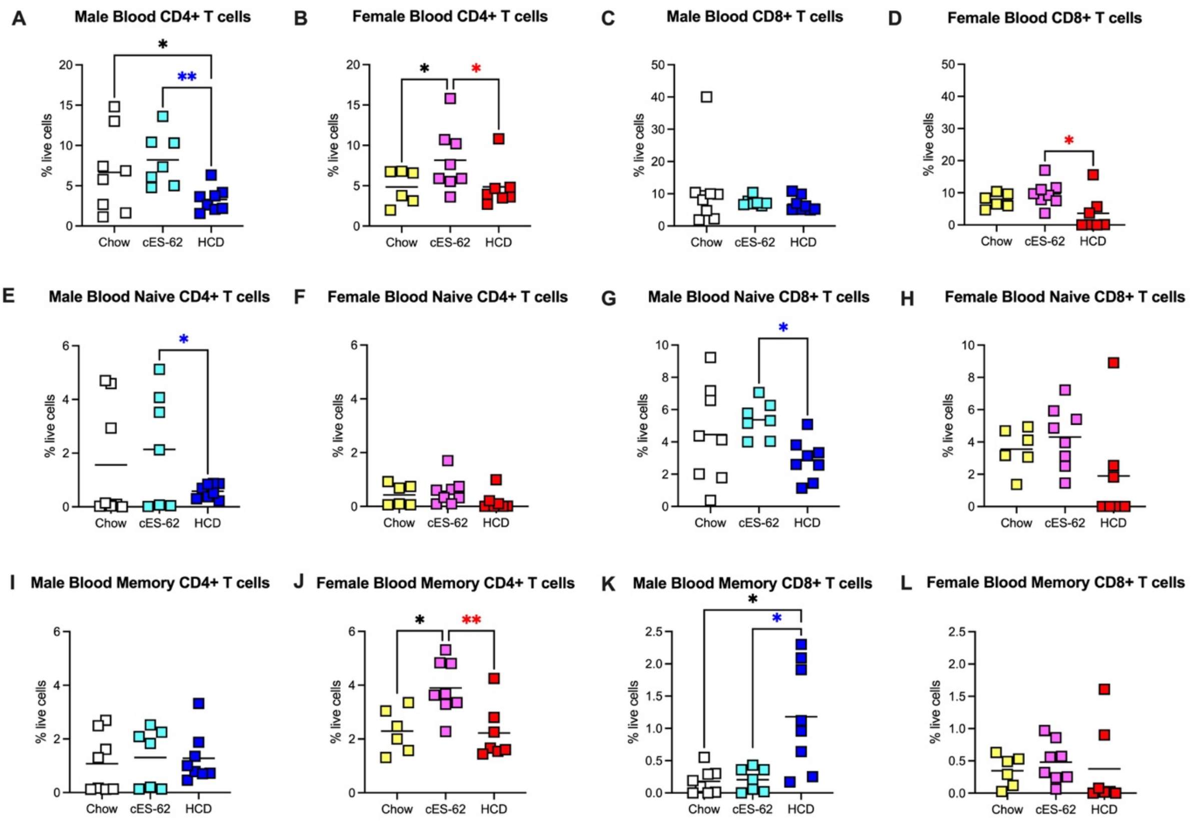

Supplementary Figure 9. Changes in the levels of splenic and MLN CD4$^+$ and CD8$^+$ T cells during obesity-accelerated ageing. The proportions of total **(A-D)** and CD103$^+$ **(E-H)** CD4$^+$ and total CD8$^+$ **(I-L)** T cells were determined at the indicated time-points as the % live cells in the spleen **(A, B, E, F, I & J)** and MLNs **(C, D, G, H, K & L)** of male **(A, C, E, G, I & K)** and female **(B, D, F, H, J & L)** mice fed Chow or HCD, the latter cohort treated with either PBS or ES-62. The data are presented as the mean ± SEM values for n individual mice in each group, where for splenic analysis of male mice, d56: n=5; d340, Chow, n=6, PBS: n=11, ES-62: n=12; d500: Chow, n=5 (3, CD8), PBS, n=6 (4, CD8), ES-62, n=6 (4, CD8) and for female mice, d56: n=5, d340: Chow, n=4, PBS, n= 11, ES-62, n=12; d500: Chow, n=3 (2, CD8), PBS, n=6 (4, CD8), ES-62, n=6 (4, CD8). For MLN analysis of male mice, d56: n=6; d340, Chow, n=4, PBS: n=7, ES-62: n=8; d500: Chow, n=4 (3, CD8), PBS, n=6 (4, CD8), ES-62, n=4 (2, CD8) and for female mice, d56: n=5, d340: Chow, n=4, PBS, n= 10, ES-62, n=10; d500: Chow, n=3 (2, CD8), PBS, n=5 (3, CD8), ES-62, n=6 (4, CD8). Significant differences are indicated by *=p < 0.05 for Chow v ES-62 and/or PBS **(B, E, I, L)**; **=p<0.01 for Chow v ES-62 and/or PBS **(E, H, L)** and ***=p<0.001 for Chow v ES-62 and/or PBS **(D, F, H, J)** groups.

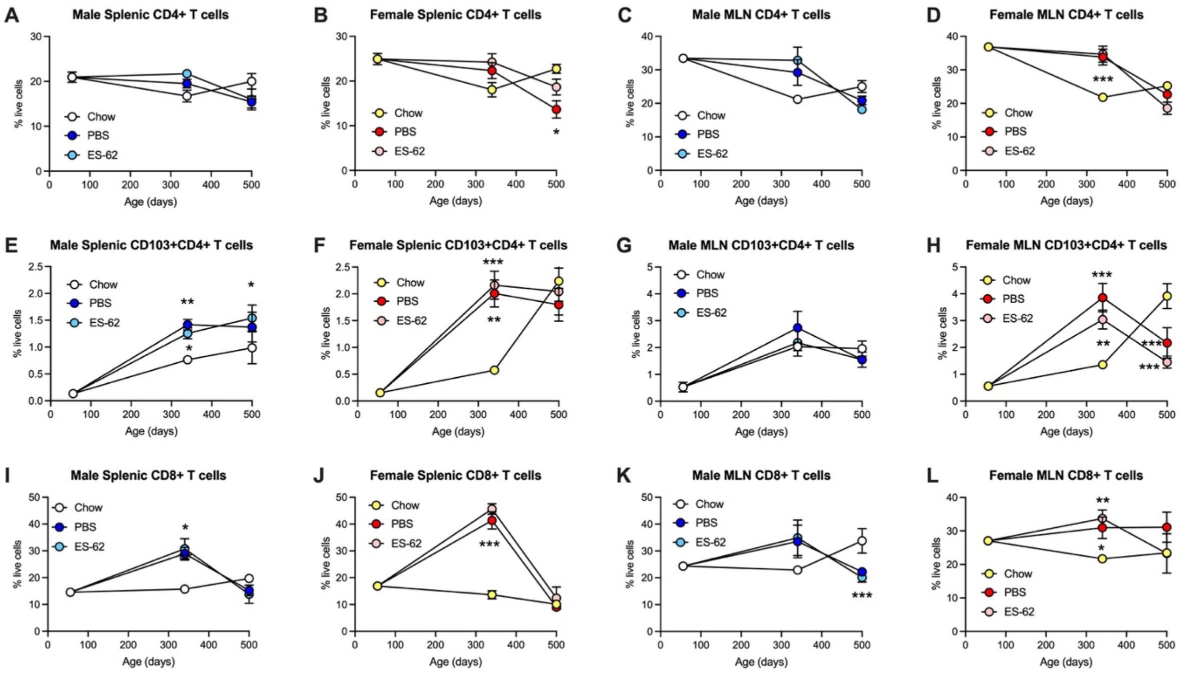

Supplementary Figure 10: HCD and SMAs do not impact on the osteoclastogenic potential of BM OCPs at d160. The levels of BM CD3^{-}B220^{-}Ter119^{-}Ly6G^{-}Ly6C^{high} CD11b^{low} OCPs were determined at d160 as the % live BM cells in **(A)** male and **(B)** female mice fed Chow or HCD, the latter cohort treated with either PBS or SMAs. (**C-F)** OC differentiation at day 5 was assessed by TRAP staining and the number and size of multinucleated OCs with >3 nuclei determined by Image J analysis. Representative images (x4 magnification) of OCs of the indicated groups of male and female mice are shown **(C)** and the mean group OC size **(D & E)** or number **(F & G)** ± SEM determined from the mean values of triplicate cultures of individual male **(D & F)** and female **(E & G)** mice at each time point. The data shown are the values of individual mice (symbols) with the mean value for the group represented by the bar.

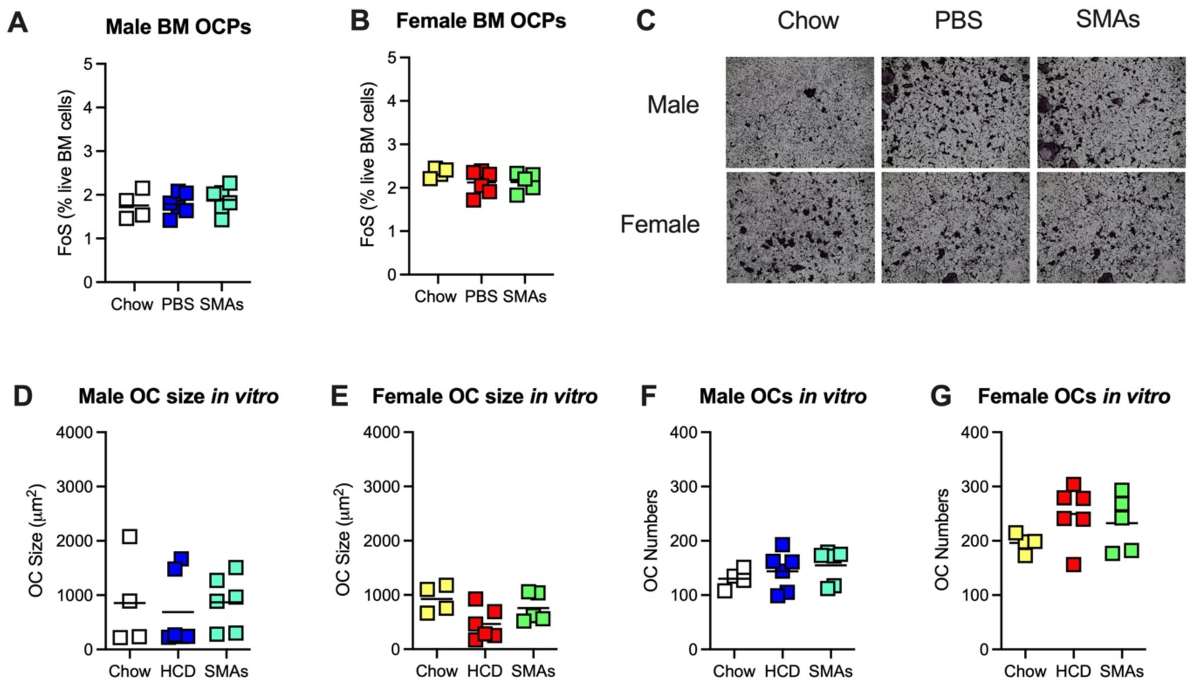

Supplementary Figure 11. SMAs do not impact on the proportions of BM cell lineages in HCD-fed mice at d160. The levels of $Lin^{-}Sca\text{-}1^{+}c\text{-}Kit^{+}$ (LSK) HSC (**A**; % Lin^{-} cells), the ratios of myeloid/lymphoid lineages (**B**) and the levels of $CD19^{+}$ B cells **(C)**, $CD3^{+}$ T cells **(D)**, $Ly6C^{+}Ly6G^{+}$ neutrophils **(E)** and $Ly6C^{+}Ly6G^{-}$ monocytes **(F)** were determined at d160 as the % live BM cells in male and female mice fed Chow or HCD, the latter cohort treated with either PBS or SMAs. **(G)** Megakaryocytes in bone sections were visualized and quantitated by Image J analysis where the mean group number/mm^2 ± SEM is determined from the mean values of triplicate field of view (FoV) of individual mice at d160. Representative images (x20 magnification, scale bars 200 µm) are shown for the indicated groups of male and female mice **(H)**. The data shown are the values of individual mice (symbols) with the mean value for the group represented by the bar and significant differences between male and female Chow groups indicated as *=p<0.05 **(C);** **=p<0.01 **(A, E)** and ***=p<0.001 **(D)**; #=p<0.05 for female Chow v female SMAs **(D)** and ##=p<0.01 for female Chow v female PBS **(D)** groups.

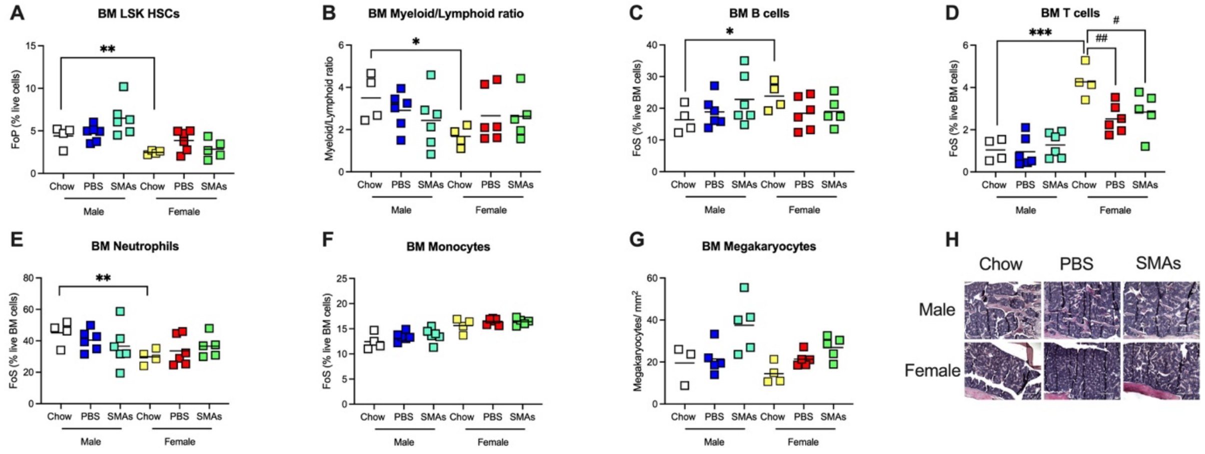

Supplementary Figure 12. Effect of HCD and SMAs on the levels of naïve and memory T cells circulating in the blood of fed mice at d160. The proportions of total CD4$^+$ **(A)** and CD8$^+$ **(B)** T cells and the levels of naïve (CD45RB$^+$CD44$^-$; **C & D**) and memory (CD45RB$^-$CD44$^+$; **E & F**) CD4$^+$ **(A, C & E)** and CD8$^+$ **(B, D & F)** T cells were determined at d160 as the % live cells in the blood of male and female mice fed Chow or HCD, the latter cohort treated with either PBS or SMAs. The data shown are the values of individual mice (symbols) with the mean value for the group represented by the bar and significant differences are indicated by *=p < 0.05 for male Chow v male SMAs and/or male PBS (**A, E, F**); **=p<0.01 for male Chow v female Chow **B, C, E)** and ***=p<0.001 for male Chow v female Chow **(A, D)** groups.

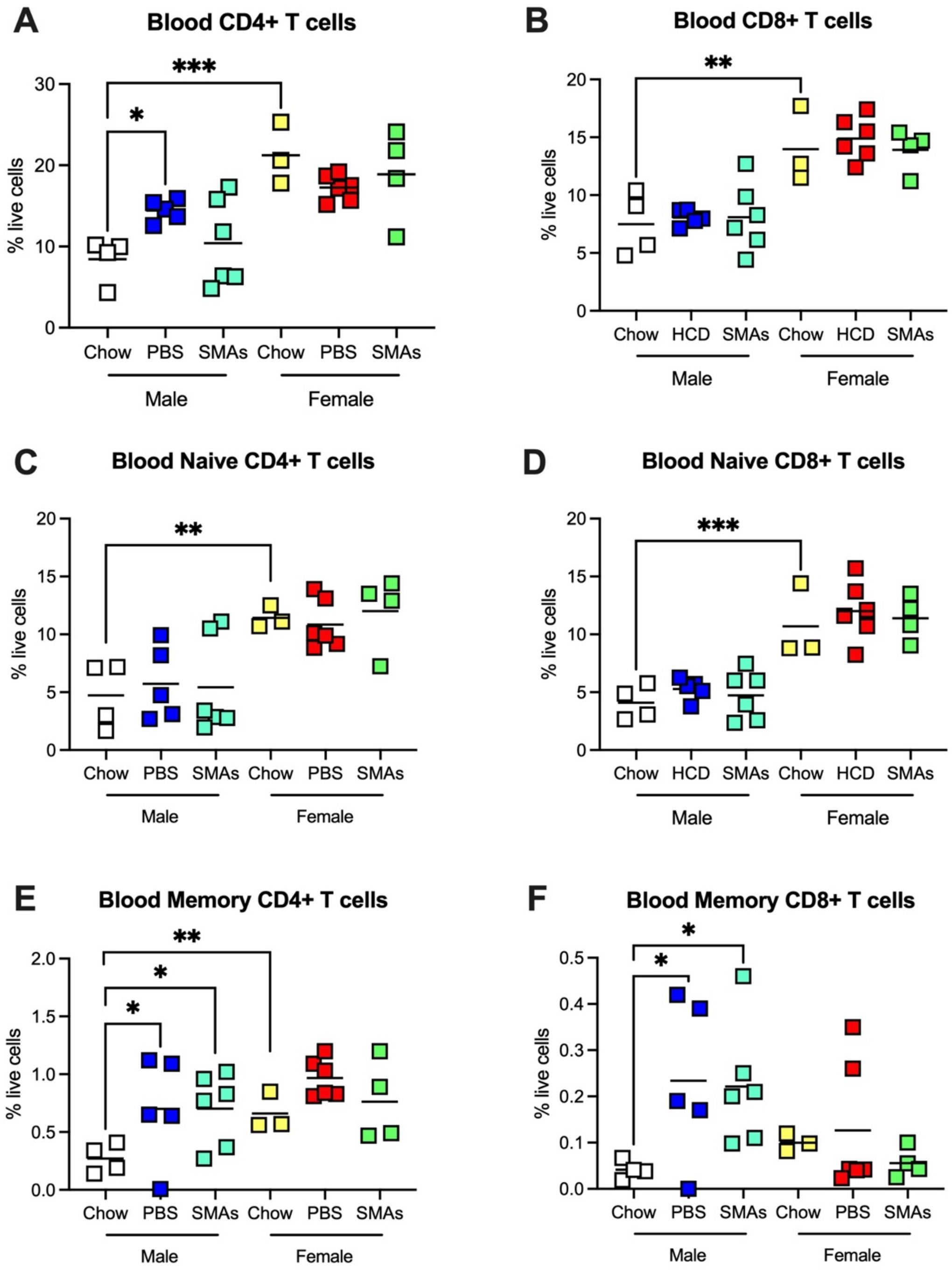

Supplementary Figure 13. Effect of HCD and SMAs on the levels of MLN and splenic T and B cell populations at d160. The levels of MLN CD4$^+$ **(A)** and CD8$^+$ **(B)** T cells, splenic CD4$^+$ **(C)** and CD8$^+$ **(D)** T cells and splenic CD19$^+$CD21$^-$CD23$^-$CD11c$^+$ ABCs **(E)** and IL-10$^+$CD19$^+$ B cells **(F)** were determined at d160 as the % live cells in spleens/MLNs of male and female mice fed Chow or HCD, the latter cohort treated with either PBS or SMAs. The data shown are the values of individual mice (symbols) with the mean value for the group represented by the bar. **(G-I)** The mRNA levels of TNFα **(G** [female data on right y-axis] **& H)** or IL-1β **(I)** in the gonadal adipose tissue of male and female mice fed Chow or HCD, the latter cohort treated with either PBS, ES-62 or SMAs as indicated. The data shown are the values of individual mice (symbols) with the mean value for the group represented by the bar. Significant differences are indicated by black**=p<0.01 for male Chow v male SMAs **(B)** or female Chow **(E, F)**; black*=p < 0.05 for male Chow v male PBS **(G-I)** groups.

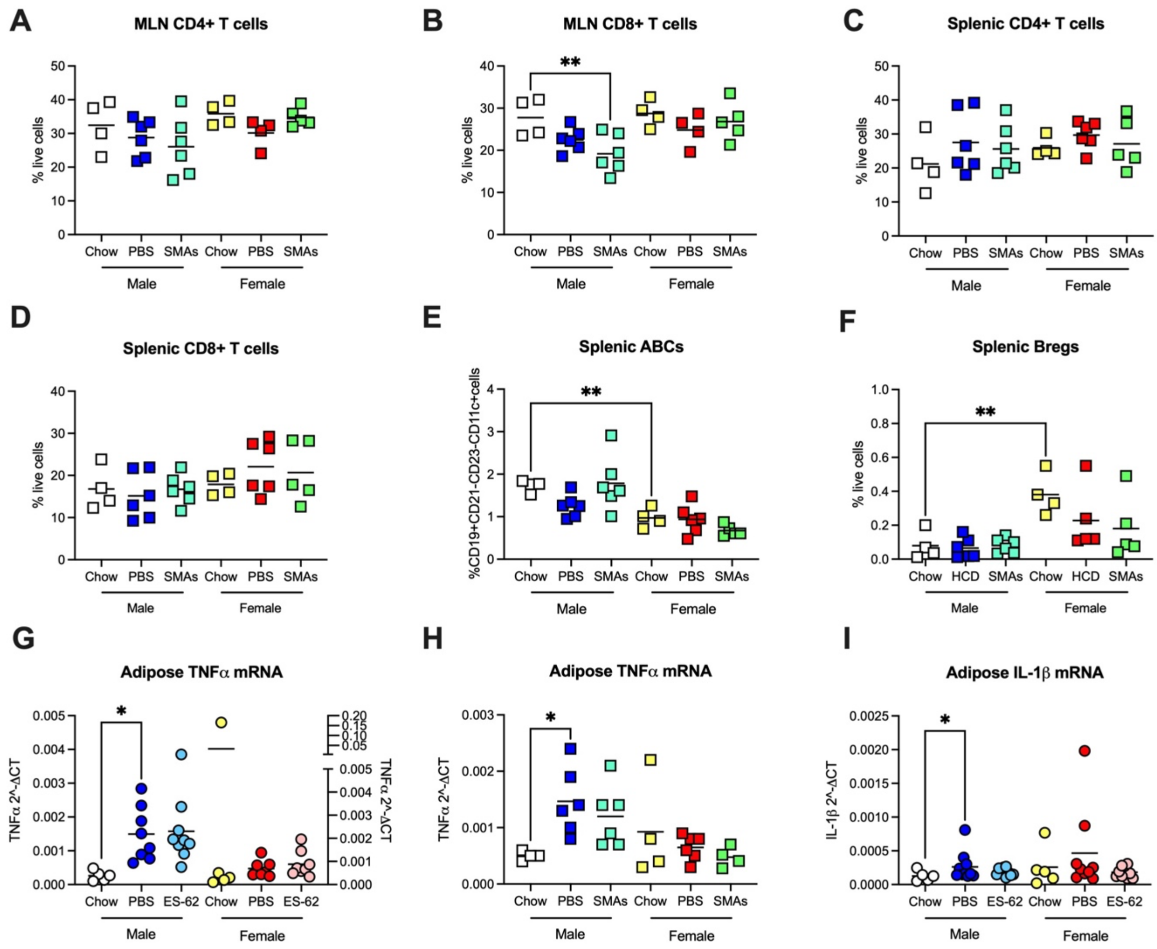

Supplement: Supplementary file 1 [file DataSheet_1.pdf]
